# Integrative transcriptome and chromatin landscape analysis reveals distinct epigenetic regulations in human memory B cells

**DOI:** 10.1038/s41467-020-19242-6

**Published:** 2020-10-28

**Authors:** Justin B. Moroney, Anusha Vasudev, Alexander Pertsemlidis, Hong Zan, Paolo Casali

**Affiliations:** 1 Department of Microbiology, Immunology & Molecular Genetics, University of Texas Long School of Medicine, UT Health Science Center, San Antonio, TX 78229 USA; 2 Greehey Children’s Cancer Research Institute, University of Texas Long School of Medicine, UT Health Science Center, San Antonio, TX 78229 USA

**Keywords:** Epigenomics, Class switch recombination, Immunological memory, B-2 cells

## Abstract

Memory B cells (MBCs) are long-lived and produce high-affinity, generally, class-switched antibodies. Here, we use a multiparameter approach involving CD27 to segregate naïve B cells (NBC), IgD^+^ unswitched (unsw)MBCs and IgG^+^ or IgA^+^ class-switched (sw)MBCs from humans of different age, sex and race. Conserved antibody variable gene expression indicates that MBCs emerge through unbiased selection from NBCs. Integrative analyses of mRNAs, miRNAs, lncRNAs, chromatin accessibility and cis-regulatory elements uncover a core mRNA-ncRNA transcriptional signature shared by IgG^+^ and IgA^+^ swMBCs and distinct from NBCs, while unswMBCs display a transitional transcriptome. Some swMBC transcriptional signature loci are accessible but not expressed in NBCs. Profiling miRNAs reveals downregulated MIR181, and concomitantly upregulated MIR181 target genes such as RASSF6, TOX, TRERF1, TRPV3 and RORα, in swMBCs. Finally, lncRNAs differentially expressed in swMBCs cluster proximal to the IgH chain locus on chromosome 14. Our findings thus provide new insights into MBC transcriptional programs and epigenetic regulation, opening new investigative avenues on these critical cell elements in human health and disease.

## Abstract (web summary)

Human memory B cells differentiate from naïve B cells and can express different immunoglobulin (Ig) isotypes resulted from class-switch recombination. Here the authors describe, using transcriptional and epigenetic data from human memory B cells and integrated multi-omics analyses, the differentiation regulation and trajectory of IgG^+^, IgA^+^ and IgD^+^ memory B cells.

## Introduction

B cell maturation in the bone marrow, peripheral differentiation, and survival is dictated by genetic programs that drive critical pathways, giving rise to plasma cells and memory B cells (MBCs). Upon activation by antigen, mature naïve B cells (NBCs) enter the germinal center (GC) reaction to undergo activation-induced deaminase (AID)-mediated somatic hypermutation (SHM) and class switch DNA recombination (CSR)^1–4^, leading to plasma cell or MBC differentiation. MBCs are central to the adaptive immune response, establishing long-term antigen-specific immunity. Their generation and function are critical for the development of anamnestic antibody responses induced by vaccines or natural infections. Effective anamnestic immune response to foreign antigens, such as those on bacteria and viruses^5^, is required for the establishment of “herd immunity” and eradication of an infectious agent in a population as a whole. MBCs generally express somatically mutated, affinity mature B cell surface receptors (BCRs) for antigen and persist in a quiescent state until re-encountering their cognate antigen^6^. While many MBCs express class-switched BCRs, such as immunoglobulin (Ig)G or IgA, “unswitched” IgD^+^IgM^+^ (unsw)MBCs also exist, with human switched (sw)MBCs and unswMBCs bearing somatically mutated BCRs and expressing surface CD27, a marker of “antigen-experience”^7–10^. Upon reactivation, MBCs rapidly proliferate and differentiate into plasma cells to secrete large amounts of high-affinity antibodies^6,11,12^. Alternatively, MBCs re-enter the GC reaction to undergo additional rounds of SHM/CSR^13,14^. Thus, enhanced responses against previously experienced antigens are contingent upon functional MBCs, which possess intrinsic advantages over NBCs, including longer lifespan and accelerated antibody response^15^.

Research to date, primarily in the mouse, has attempted to attribute intrinsic differences between MBCs and NBCs to select gene expression programs in MBC differentiation, survival, and reactivation^16–21^. IL-9 signaling has been suggested to drive MBC development within GCs as well as mediate antibody recall responses^16,17^. High Bach2 expression biases GC B cells towards MBC differentiation, while reduced Bach2 expression skews MBCs towards plasma cell differentiation upon reactivation^18,19^. Divergent transcriptional programming has been tentatively identified in MBCs, with mouse IgG2a^+^ and IgA^+^MBCs utilizing T-bet and Rorα, respectively, to maintain their distinct identities and functions^20^. As recently reported, the transcription factor (TF) Hhex interacts with the corepressor Tle3 to promote MBC differentiation, but not maintenance of mouse MBCs^22^. Finally, MBCs accelerated and heightened response has been attributed to reduced expression of quiescence factors, such as *ZBTB16*, *KLF4,* and *KLF9*, as well as enhanced intrinsic signaling of class-switched BCR Igγ chain. This carries tail tyrosine ITT motifs that reduce IgG^+^MBCs activation threshold^21,23^.

The human MBC transcriptome and its regulation, particularly by epigenetic mechanisms, need better understanding^24,25^. As we and others have shown, coordinated regulation of gene networks is critical in human and mouse B cell differentiation and antibody responses^26–33^. Such coordinated regulation is mediated by epigenetic modifications and factors, including DNA methylation, histone post-translational modifications, and non-coding RNAs (ncRNAs), such as microRNAs (miRNAs) and long non-coding RNAs (lncRNAs)^34,35^. These layers of epigenetic regulation synergize with TFs and chromatin accessibility to outline distinct gene expression programs, thereby dictating cell functions^26,36^. Although different miRNA profiles have been reported in different B cell subsets, including MBCs^37^, and lncRNAs have been associated with different stages of B cell development^38^, no comprehensive analysis of the protein-coding and non-coding transcriptome and chromatin accessibility has been reported in human MBCs.

Integrative analysis of differentially expressed mRNAs, miRNAs, lncRNAs, chromatin landscape, and *cis*-regulatory elements reveals how gene expression intersects with epigenetic regulation to segregate distinct MBCs. Analysis of CD27^–^IgD^+^ NBCs, CD27^+^IgD^+^ unswMBCs, CD27^+^IgG^+^ swMBCs and CD27^+^IgA^+^ swMBCs as well as total CD27^−^ NBCs and CD27^+^ MBCs from healthy human subjects defines a differential transcriptional signature that distinguishes CD27^+^IgG^+^ swMBCs and CD27^+^IgA^+^ swMBCs from CD27^–^IgD^+^ NBCs, stratifying CD27^+^IgD^+^ unswMBCs between the two. The integration of RNA-Seq with ATAC-Seq data uncovers distinct profiles of *cis*-regulatory elements in human MBCs. MIR181 downregulation with concomitant upregulation of this microRNA’s target genes indicates an important role for MIR181 in MBC function(s). Further, the inverse correlation of upregulated MIR181 sponge, lncRNA *MIAT,* with downregulated MIR181a/MIR181b in swMBCs points to *MIAT* as a regulator that, along with chromatin accessibility, reduces MIR181 expression and promotes swMBC-specific gene expression. Overall, our findings provide evidence for overlapping layers of regulation, including chromatin remodeling, *cis-*regulatory elements, and distinct sets of miRNAs and lncRNAs, which integrate to dictate gene expression profiles and activation pathways characteristic of human MBCs.

## Results

### Identification and purification of human swMBCs, unswMBCs, and NBCs

**Fig. 1:**
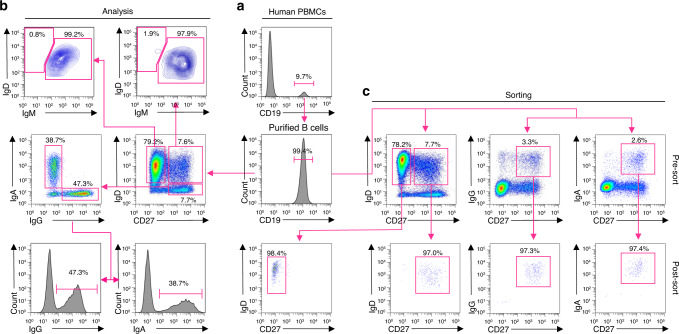
Identification and purification of CD27^−^IgD^+^, CD27^+^IgD^+^, CD27^+^IgG^+^, and CD27^+^IgA^+^ B cells. **a** Purification of CD19^+^ B cells from PBMCs by immunomagnetic negative selection. **b** FACS analysis of CD19^+^ B cells for surface expression of CD27, IgD, IgM, IgG, and IgA. **c** FACS sorting of CD19^+^ cells (subjects B, C. and G) into four subsets: CD27^−^IgD^+^ NBCs, CD27^+^IgD^+ ^unswMBCs, CD27^+^IgG^+^ swMBCs, and CD27^+^IgA^+^ swMBCs using the indicated gating strategy and two distinct staining panels. Three independent experiments. The purity of the sorted four B cell subsets as analyzed by FACS. Data are from subject G.

To elucidate the transcriptional landscape of human MBCs, we set up to identify and isolate MBCs and their NBC counterparts. We purified B cells (>99% CD19^+^) from PBMCs of 7 healthy human subjects (age 20–39, 4 males, 3 females of different race and ethnic backgrounds) (Supplementary Table 1) by immunomagnetic negative selection (Fig. 1a). CD19^+^ cells were analyzed for surface expression of CD27, a marker for antigen-experienced B cells, as well as IgD, IgM, IgG, and IgA. This allowed for the identification of four distinct subsets: CD27^–^IgD^+^ NBCs (63.9 ± 14.3%), CD27^+^IgD^+^ unswMBCs (9.7 ± 8.3%), CD27^+^IgG^+^ swMBCs (6.5 ± 3.1%) and CD27^+^IgA^+^ swMBCs (4.9 ± 2.0%). A fifth subset identified “double negative” CD27^–^IgD^–^ accounted for the remaining 15.0% of B cells. CD27^–^ B cells were IgD^+^IgM^+^, while CD27^+ ^B cells comprised comparable proportions of IgD^+^ and IgD^–^ B cells. CD27^+^IgD^+^ B cells were mostly IgM^+^ with only 3.8 ± 3.0% IgD^+^IgM^–^ B cells. CD27^+^IgD^–^ B cells were primarily either IgG^+^ or IgA^+^ (Fig. 1b). CD19^+^ cells from subjects B, C and G were sorted into 4 fractions: CD27^–^IgD^+^ NBCs (70.2 ± 15.2%), CD27^+^IgD^+^ unswMBCs (10.1 ± 5.1%), CD27^+^IgG^+^ swMBCs (6.6 ± 5.2%) and CD27^+^IgA^+^ swMBCs (4.3 ± 2.7%). Reanalysis of the sorted fractions confirmed the identity and purity of the 4 discrete B cell subsets (Fig. 1c). Finally, CD19^+^ cells from the remaining four subjects (A, D, E, and F) were analyzed and isolated as CD27^−^ (NBCs) and CD27^+^ total (MBCs) (Supplementary Table 1).

### IgV_H_, J_H_, and V_L_, J_L_ gene expression in human NBCs and MBCs

**Fig. 2:**
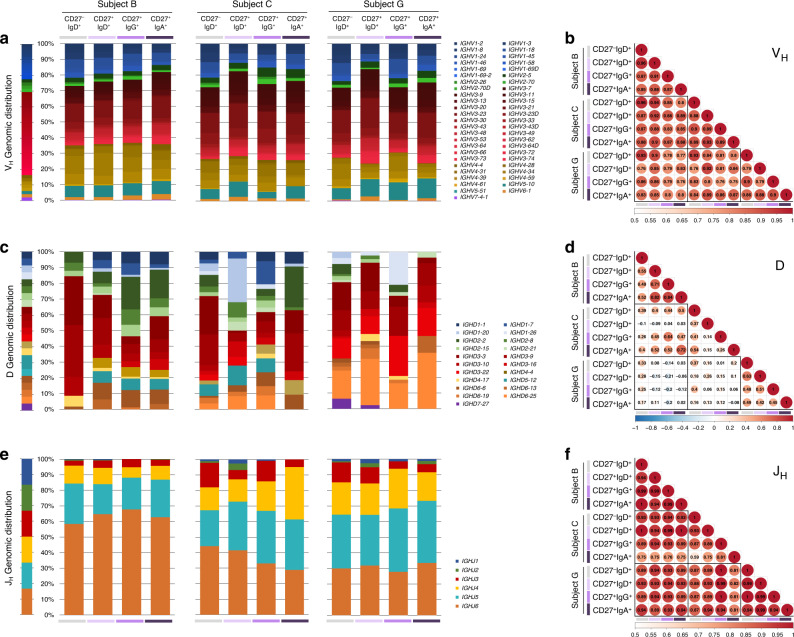
Expressed Ig V_H_, D, and JH repertoire in human CD27^−^IgD^+^, CD27^+^IgD^+^, CD27^+^IgG^+^, and CD27^+^IgA^+^ B cells. **a**, **c**, **e** Ig V_H_ (**a**), D (**c**), and J_H_ (**e**) gene expression in CD27^−^IgD^+^, CD27^+^IgD^+^, CD27^+^IgG^+^, and CD27^+^IgA^+^ B cells, depicted by stacked column for each subject (subjects B, C, G). Ig gene family members grouped within each Ig V_H_, D, J_H_ family or subgroup are depicted in shades of the respective family/group color. V_H_ family: V_H_1 (blue), V_H_2 (green), V_H_3 (red), V_H_4 (yellow), V_H_5 (teal), V_H_6 (orange), V_H_7 (purple). D family: D1 (blue), D2 (green), D3 (red), D4 (yellow), D5 (teal), D6 (orange), D7 (purple). J_H_ family: J_H_1 (blue), J_H_2 (green), J_H_3 (red), J_H_4 (yellow), J_H_5 (teal), J_H_6 (orange). **b**, **d**, **f** Correlation matrix showing the relationship of individual Ig V_H_ (**b**), D (**d**), and J_H_ (**f**) gene expression between B cell subsets in the three subjects analyzed. Color bars depict the correlation coefficients on a scale from 0.5 (white) to 1 (red) (**b**, **f**), or from −1 (blue) to 0 (white) to 1 (red) (**d**), with color, circle size, and value all indicating the overall strength of the correlation. B cell subsets are repeated at the bottom, left to right in CD27^−^IgD^+^ (gray), CD27^+^IgD^+^ (lavender), CD27^+^IgG^+^ (purple), and CD27^+^IgA^+^ (dark purple).

We analyzed the expression of Ig V_H_, D, J_H_, and Vκ, Jκ, as well as Vλ, Jλ genes in CD27^–^IgD^+^ NBCs, CD27^+^IgD^+^ unswMBCs, CD27^+^IgG^+^ swMBCs, and CD27^+^IgA^+^ swMBCs. The human IgH locus comprises 36–49 functional V_H_ genes segregated into 7 families^39^. In all four subsets within each subject, V_H_ family expression was stochastic (*r*_s_ = 0.98), reflecting the genomic representation of the 7 V_H_ families, with V_H_3 genes being the most represented (37.8-59.9%), followed by V_H_4 (9.7–26.4%) and V_H_1 (11.3–23.7%) (Fig. 2a). Expression of individual V_H_ genes was conserved in each V_H_ family, across the 4 subsets in each subject (*r*_s_ = 0.88) and across the three subjects (*r*_s_ = 0.84) (Fig. 2b). As expected, D gene expression was diverse across the four subsets in each subject (*r*_s_ = 0.49) and across the three subjects (*r*_s_ = 0.17) (Fig. 2c, d). J_H_ gene usage was conserved across the four subsets within each subject (*r*_s_ = 0.90), with a preponderant J_H_5 (19.2–40.5%) and J_H_6 (27.8–67.7%) utilization in all three subjects, albeit to differing degrees (Fig. 2e, f).

The human Igκ locus comprises 39 functional Vκ and 5 Jκ genes^39^. Vκ and Jκ gene expression were comparable across the four subsets within each subject (*r*_s_ = 0.88; 0.86, respectively) and across the three subjects (*r*_s_ = 0.86; 0.82, respectively). Vκ gene usage was biased towards Vκ3 and Vκ4 and away from Vκ1 and Vκ2. Jκ gene usage was biased towards Jκ1, Jκ3, and Jκ5 (Supplementary Fig. 1a–d). The human Igλ locus comprises 30 functional Vλ genes segregated into 10 subgroups and 5 functional Jλ-Cλ clusters^39^. Vλ gene expression was comparable across the four subsets within each subject (r_s_ = 0.82) and across the three subjects (*r*_s_ = 0.83), with over-representation of Vλ3, Vλ2, and Vλ1 subgroups (Supplementary Fig. 1e, f). Jλ-Cλ usage was biased towards Jλ3-Cλ3 (35.2–57.5%) and Jλ2-Cλ2 (17.9–54.6%), with conserved distributions in all four subsets (*r*_s_ = 0.87) (Supplementary Fig. 1g, h).

### IgH CDR3 length and somatic point-mutations in human MBCs

**Fig. 3:**
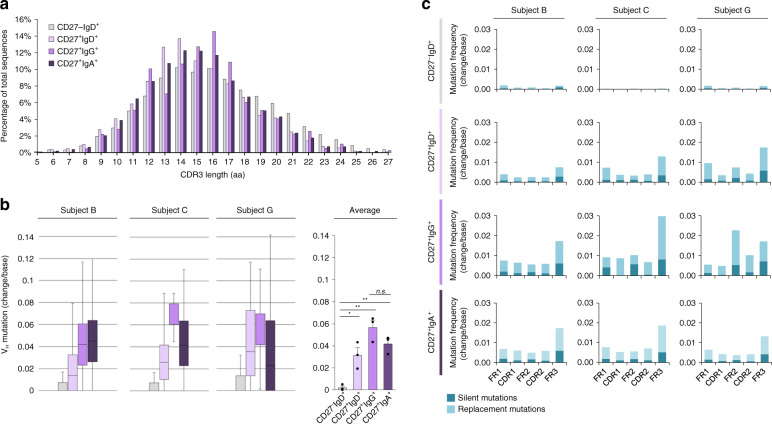
CDR3 lengths and somatic point-mutations in recombined *Ig* V_H_DJ_H_ gene segments of human MBCs. **a** Average percentage of total sequences at any given CDR3 length in recombined V_H_DJ_H_ transcripts expressed by CD27^–^IgD^+^, CD27^+^IgD^+^, CD27^+^IgG^+^, and CD27^+^IgA^+^ B cells. **b** Somatic point-mutations in recombined Ig V_H_DJ_H_ transcripts expressed by CD27^–^IgD^+^ (4302 transcripts), CD27^+^IgD^+^ (4705 transcripts), CD27^+^IgG^+^ (1564 transcripts), and CD27^+^IgA^+^ (13,549 transcripts) B cells with boxplots depicting the frequencies of point-mutations (change/base). Data are represented as boxplots where the middle line is the median, the lower and upper hinges correspond to the first and third quartiles, the upper whisker extends from the hinge to the largest value no further than 1.5 × IQR from the hinge (where IQR is the inter-quartile range) and the lower whisker extends from the hinge to the smallest value at most 1.5 × IQR of the hinge. *p < 0.05, **p < 0.01, ***p < 0.001, ns not significant (paired two-sided *t-*test). **c** Frequency of silent and repla**c**ement point-mutations in framework regions (FR) and complementarity-determining regions (CDRs) in all four sorted B cell subsets for each of the three healthy human subjects. Data are mean ± SEM of three independent experiments. B cell subsets are repeated at the bottom, left to right in CD27^−^IgD^+^ (gray), CD27^+^IgD^+^ (lavender), CD27^+^IgG^+^ (purple), and CD27^+^IgA^+^ (dark purple).

Somatic *IgH* V_H_DJ_H_ rearrangement determines the sequence and length of the complementary determining region 3 (CDR3), which is critical for BCR-antigen contact. To address IgH CDR3 length and nature as well as V_H_ mutational load, swMBCs, unswMBCs, and NBCs, the recombined V_H_DJ_H_-C_H_ transcripts were amplified using forward (degenerate) primers for V_H_1, V_H_2, V_H_3, V_H_4, V_H_5, V_H_6, V_H_7 genes leader sequences in conjunction with reverse Cμ, Cδ, Cγ, or Cα isotype-specific primer and analyzed by IMGT/HighV-QUEST^40^. The distribution of CDR3 lengths was largely conserved among NBCs (16.05 ± 0.10 AA’s), unswMBCs (14.86 ± 0.11 AA’s), IgG^+^ swMBCs (15.53 ± 0.12 AA’s), and IgA^+^ swMBCs (15.08 ± 0.11 AA’s) (Fig. 3a). A salient feature of MBCs is the load of somatic point-mutations in expressed *Ig* V(D)J genes, a result of precursor B cells undergoing antigen-driven SHM and selection^41^. Consistent with their non-antigen-experienced status (CD27^−^), NBCs exhibited a negligible frequency of somatic point-mutations (0.0020 change/base). By contrast, consistent with their antigen-experienced status, CD27^+^ B cells, be they IgG^+^, IgA^+^ or IgD^+^ exhibited a higher frequency of somatic point-mutations (0.0314, 0.0566 and 0.0417 change/base, respectively), mostly replacement mutations, than NBCs (Fig. 3b, c), with IgG^+^ swMBCs and IgA^+^ swMBCs carrying greater mutational burdens than unswMBCs.

### A core transcriptional signature distinguishes IgG^+^ swMBCs and IgA^+^ swMBCs from NBCs

**Fig. 4:**
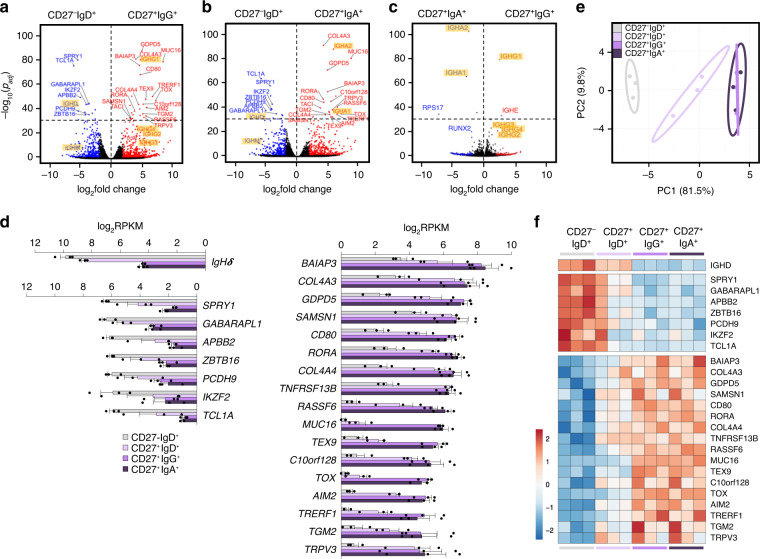
Transcriptome analysis in human NBCs and swMBCs. **a**–**c** Global transcriptional differences of mRNAs in CD27^+^IgG^+^ vs. CD27^–^IgD^+^ B cells (**a**), CD27^+^IgA^+^ vs. CD27^–^IgD^+^ B cells (**b**) and CD27^+^IgG^+^ vs. CD27^+^IgA^+^ B cells (**c**), as depicted by volcano plots. All genes annotated in human GENCODE v24 GRCh38 are shown, with each circle representing 1 mRNA. The −log_10_ FDR-adjusted *p* (*p*_adj_) value is shown on the *y* axis (*p*_adj_ < 10 × 10^–30^ indicated above-dashed line). DE mRNAs at *p*_adj_ < 0.05 are highlighted in red (upregulated) or in blue (downregulated). DE mRNAs at *p*_adj_ < 10 × 10^–30^ and common to both CD27^+^IgG^+^ and CD27^+^IgA^+^ MBCs are annotated; *IgH* chain transcripts are annotated and highlighted. **d** Normalized (log_2_RPKM) expression of the 24 (IgHδ excluded) DE mRNAs at *p*_adj_ < 10 × 10^–30^ in swMBCs as compared to NBCs depicted by histogram for each B cell subset (left, downregulated and right, upregulated). Data are mean ± SEM from all three subjects. **e** Transcriptome clustering of the sorted subsets performed using the top 24 DE mRNAs depicted by principal component analysis (PCA) plot. Prediction ellipses define 95% confidence intervals. Each symbol represents an individually sorted subset (*n* = 3). **f** Relative expression profiles of swMBC core transcriptional signature mRNAs at *p*_adj_ < 10 × 10^–30^ compared by heatmap, depicting relative transcriptional changes across CD27^–^IgD^+^, CD27^+^IgD^+^, CD27^+^IgG^+^, and CD27^+^IgA^+^ B cell subsets in each subject (order^;^ B^,^C,G). Data in **a**–**f** depict DE mRNA as determined by edgeR. B cell subsets are repeated at the bottom, left to right in CD27^–^IgD^+^ (gray), CD27^+^IgD^+^ (lavender), CD27^+^IgG^+^ (purple), and CD27^+^IgA^+^ (dark purple).

Using next-generation sequencing, we identified differentially expressed (DE) mRNA transcripts in CD27^+^IgG^+^ vs. CD27^–^IgD^+^ B cells and CD27^+^IgA^+^ vs. CD27^–^IgD^+^ B cells by pairwise comparisons. *IgCγ* and *IgCα* transcripts were significantly increased and *IgCμ* and *IgCδ* correspondingly decreased in CD27^+^IgG^+^ and CD27^+^IgA^+^ swMBCs (Fig. 4a, b). The higher *IgCγ1* and *IgCα2* expression reflected the peripheral blood predominance of IgG1 and IgA2 subclasses and respective B cells. Analysis at the highest level of significance of *p*_adj_ < 10 × 10^–30^ identified the same 24 (17 upregulated and 7 downregulated) DE mRNAs (not including *IgC*δ) in both IgG^+^ and IgA^+^ swMBCs as compared to their NBC counterparts (Fig. 4a, b and Supplementary Fig. 2a, b, Supplementary Table 2). *RSP17*, a ribosomal subunit protein, and *RUNX2*, a TF essential for CSR to IgA, were highly expressed in IgA^+^ swMBCs as compared to IgG^+^ swMBCs (Fig. 4c). *IgCε* expression in IgG^+^ swMBCs, but not IgA^+^ swMBCs, likely reflected the sequential CSR poise from IgG1 to IgE of such swMBCs^42^. Importantly, the “cancellation” of the 24 DE genes when comparing CD27^+^IgG^+^ vs. CD27^+^IgA^+^ B cells outlined a gene expression profile characteristic and distinctive of swMBCs, regardless of BCR isotype (Fig. 4c and Supplementary Table 2). This was strengthened by an equivalent level of normalized expression of the 24 transcripts in IgG^+^ swMBCs and IgA^+^swMBCs (Fig. 4d).

The transcriptional distance between the four B cell subsets, as measured by principal component analysis (PCA), yielded three discrete clusters, identifying NBCs, unswMBCs, and swMBCs (Fig. 4e and Supplementary Fig. 2c). The 24 DE genes discriminated IgG^+^ swMBCs and IgA^+^swMBCs from the other two B cell subsets across all three subjects (Fig. 4f). Further, the 24 DE genes discriminated total MBCs (CD27^+^ B cells) from NBCs (CD27^−^ B cells) in the 4 additional healthy subjects (A, D, E, F) (Supplementary Table 1 and Supplementary Fig. 2c, d). Thus, at the highest level of significance, 24 DE mRNAs characteristically distinguish class-switched IgG^+^ swMBCs and IgA^+^ swMBCs from their NBC counterparts (swMBC core transcriptional signature). 

### Gene expression outlines distinct signaling and TF activities in swMBCs

**Fig. 5:**
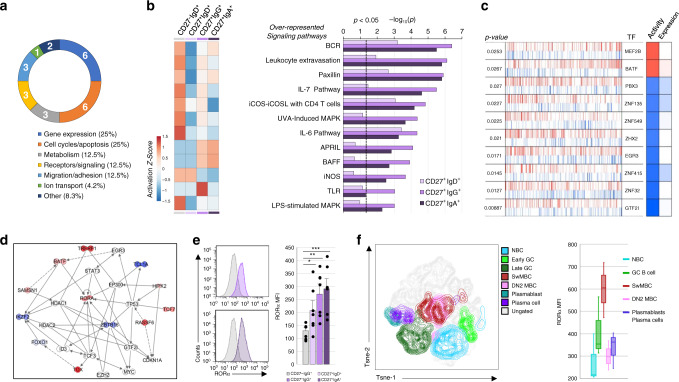
Functional characterizations of the swMBC core transcriptional signature. **a** Functional gene ontology annotation of the 24 DE genes at *p*_adj_ < 10 × 10^−^^30^ in swMBCs vs. NBCs (swMBC core transcriptional signature). Numbers denote genes in each category. **b** Canonical pathways in swMBCs and unswMBCs displaying significant increases or decreases in activation status were determined by IPA, with −log_10_-transformed *p* shown on the *x* axis (*p* < 0.05 indicated by the dashed line). Activation *Z*-scores of pathways in each subset are shown by heatmap color-coded according to legend: increased activation, red; decreased activation, blue. **c** SwMBC transcriptional regulators as identified by the MARINa algorithm, which is measured using as differentially expressed target odds ratio^90^. Gene expression is rank-ordered from the most downregulated to the most upregulated gene in swMBCs. In each row, the transcription factor (TF) names are given; positive (activated; red bars) and negative (repressed; blue bars) targets of the regulator have plotted along with the gene expression signature in rank order. The first column on the right depicts inferred differential activity of the regulator (Activity), while the second shows the regulator differential expression (expression). **d** IPA network analysis indicating annotated interactions between MARINa-inferred TFs, nucleus-localized DE swMBC signature genes, and IPA-identified cofactors. CD27^+^IgG^+^ and CD27^+^IgA^+^ B cell data sets are overlaid on the IPA network, depicting upregulated genes in red and downregulated genes in blue. **e** RORα expression in CD27^-^IgD^+^, CD27^+^IgD^+^, CD27^+^IgG^+^, and CD27^+^IgA^+^ B cells from peripheral blood of seven healthy human donors, as analyzed by intracellular staining followed flow cytometry. *p < 0.05, **p < 0.01, ***p < 0.001, ns not significant (paired two-sided *t-*test). Data are mean ± SEM of three independent experiments. **f** RORα expression in B cell fractions from the tonsil of three human subjects, as analyzed by intracellular staining followed flow cytometry. Fractions analyzed comprise NBCs (CD19^+^CD38^−^CD27^−^IgD^+^CD138^−^; blue), GC B cells (CD19^+^CD38^lo^CD27^+/−^IgD^+/−^CD138^−^; green), swMBCs (CD19^+^CD38^−^CD27^+^IgD^−^CD138^−^; red), DN2 MBCs (CD19^+^CD38^−^CD27^−^IgD^−^CD138^−^; pink), plasmablasts and plasma cells ^(^CD19^+/−^CD38^hi^CD27^+^IgD^−^CD138^+/−^; purple). Data are represented as boxplots where the middle line is the median, the lower and upper hinges correspond to the first and third quartiles, the upper whisker extends from the hinge to the largest value no further than 1.5 × IQR from the hinge (where IQR is the inter-quartile range) and the lower whisker extends from the hinge to the smallest value at most 1.5 × IQR of the hinge. Quantification of RORα MFI in **e** (*n* = 7) and **f** (*n* = 3). B cell subsets; CD27^–^IgD^+^ (gray), CD27^+^IgD^+^ (lavender), CD27^+^IgG^+^ (purple), and CD27^+^IgA^+^ (dark purple).

Among the 24 swMBC core transcriptional signature genes, six have a role in cell cycle and apoptosis, and six are implicated in gene expression (Fig. 5a). The aggregate mRNA profile (DE transcripts, *p*_adj_ < 0.05) in MBCs was explored by IPA and revealed significant over-representation in leukocyte extravasation as well as cytokine and MAP kinase signaling pathways. The activation status of such pathways differed significantly between swMBCs and NBCs (*Z*-score) (Fig. 5b), suggesting a contribution of such pathways to MBC function. As mRNA expression fails to capture post-transcriptional and post-translational events, we inferred the differential activity of TFs in human MBCs by applying a master regulator inference algorithm (MARINa) using a human B cell-specific Bayesian interactome^31^. In swMBCs, MEF2B and BATF were inferred to have increased activity, while GTF2I, EGR3, and multiple zinc-finger TFs were inferred to have decreased activity (Fig. 5c). Two DE genes, RORα, and ZBTB16, were central to a transcriptional network that incorporated MARINa-inferred TFs, nucleus-localized swMBC core transcriptional signature genes, and IPA-identified coactivators and corepressors (Fig. 5d). Probing deeper into MARINa TF analysis, ZBTB16 was found to be significant (*p* = 0.034), ranking 15th out of the 621 TFs in the regulon. RORα was not part of the transcriptional “regulon” object^31^ and, consequently, its activity was not addressed here. Analysis of RORα protein, however, showed it to be significantly expressed in swMBCs and unswMBCs across the seven human subjects (Fig. 5e). Further, RORα protein was analyzed in five discrete tonsil B cell subsets from three additional subjects and was highly expressed in such tonsil swMBCs (Fig. 5f). Thus, our integrative analysis identified signaling pathways and TF networks that are important to MBC identity and/or functions, suggesting a central role for TF RORα in the phenotype/function of human swMBCs.

### unswMBCs display a transcriptional profile of transition between NBCs and swMBCs

Although the memory phenotype of unswMBC is an accepted notion, the nature of these CD27^+^IgD^+^ B cells remains a matter of debate. Virtually all CD27^+^IgD^+^ B cells retained expression of surface IgM (Fig. 1b), yet they were reported to express mutated Ig V_H_ genes^8^. These unswMBCs carried significantly more somatic point-mutations than NBCs (Fig. 3b, c) and displayed (pairwise comparison) two DE upregulated transcripts, *TFEC* and *ZBTB32*, at *p*_adj_ < 10 × 10^–30^, as well as four swMBC core signature transcripts (*TRPV3*, *TACI*, *COL4A4*, and *SAMSN1*), upregulated at a significance of *p*_adj_ < 10 × 10^–15^ (Supplementary Fig. 3a, b and Fig. 4d). PCA showed unswMBCs clustering between NBCs and swMBCs (Fig. 4e) and displayed at lower levels signaling pathways characteristic swMBCs (Fig. 5b). PCA and hierarchical clustering analyses were expanded across the DE gene profile at differing levels of significance and consistently showed CD27^+^IgD^+^ unswMBCs as an independent cluster, slightly closer to swMBCs (Supplementary Fig. 4). Overall, CD27^+^IgD^+^ unswMBCs display a transcriptome of transition from NBCs to swMBCs.

### Chromatin accessibility and active gene landscape in MBCs and NBCs

**Fig. 6:**
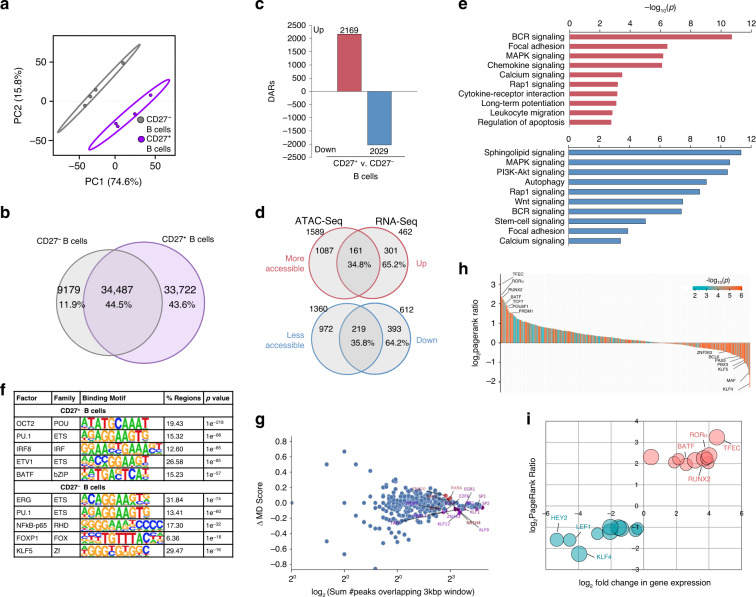
The chromatin landscape of human MBCs. **a** Clustering of CD27^–^ NBCs and CD27^+^ total MBCs based on top differentially accessible loci as displayed by PCA (4198). Prediction ellipses define 95% confidence intervals. Each symbol represents an individually sorted subset (*n* = 4). **b** Overlap of accessible loci between NBCs and total MBCs depicted by Venn diagram. **c** Differential chromatin accessibility (*p* < 0.05) between total MBCs and NBCs as determined by DEseq2. Differentially accessible regions (DARs) with increased (red) or decreased (blue) accessibility depicted by histograms. **d** Proportion of DE genes that overlap with DARs, as depicted by Venn diagrams. Percentage of genes exhibiting corresponding increases/decreases in chromatin accessibility shown below the number of genes. **e** Statistically over-represented KEGG pathways associated with increased (red) or decreased (blue) DARs depicted by the histogram. **f** Significant TF- binding motifs enriched in NBC- or all MBC-specific open chromatin regions as determined by HOMER motif analysis. **g** For all motifs (dots), the changes in MD score between NBCs and total MBCs (y-axis) are (MA) plotted against the number of motifs within 1.5 kb of any ATAC-Seq peak center (*x* axis)—computed by DAStk^94^. Red points depict statistically significant increased MD-scores (*p* < 0.05). **h**, **i** RNA-Seq and ATAC-Seq data sets were integrated using the Taiji PageRank algorithm^92^ to generate PageRank activity scores and *p*-values for human TFs. **h** All 421 TFs (*p* < 0.05) plotted in rank order according to their log_2_PageRank score ratio between MBCs vs. NBCs. Each TF PageRank −log_10_(*p)* is denoted by color-coding according to legend: more significance, red; less significance, blue. **i** The top 10 TFs in NBCs and top 10 TFs in MBCs was plotted according to their log_2_PageRank score ratio and log_2_fold change in transcript expression in MBCs vs. NBCs. The bubble size of each TF is determined by the −log_10_(*p*) of Taiji analysis.

As RNA expression is dependent upon DNA accessibility, we sought to define the chromatin landscape of human MBCs by ATAC-Seq and characterize its intersection with the transcriptome in these B cells (subjects A, D, E, F). Of the 77,388 accessible loci identified, NBCs (CD27^−^ B cells) and total MBCs (CD27^+^ B cells) displayed distinct patterns of chromatin accessibility, as indicated by component 1 of PCA (74.6% variance) (Fig. 6a). Overall, total MBCs trended towards higher chromatin accessibly, with 43.6% of identified peaks unique to them, 11.9% of peaks unique to NBCs, and 44.5% of peaks accessible in both total MBCs and NBCs (Fig. 6b). Differentially accessible regions (DARs) at *p* < 0.05 were identified in total MBCs vs. NBCs. Overall, 4198 loci were differentially accessible in total MBCs vs. NBCs, with 2169 (51.7%) DARs with increased accessibility and 2029 (48.3%) with decreased accessibility (Fig. 6c).

To determine whether cell-specific open chromatin regions identified by ATAC-Seq correlated with cell-specific gene expression, we integrated our NBC and MBC ATAC-Seq and mRNA-Seq data sets. Overall, 462 and 612 DE mRNAs were at significantly higher or lower levels, respectively, in both IgG^+^swMBCs and IgA^+^ swMBCs compared to NBCs at *p*_adj_ < 0.05. Of the 462 upregulated genes, 161 displayed increased chromatin accessibility nearby or within their loci. Of the 612 downregulated genes, 219 displayed decreased chromatin accessibility nearby and/or within their loci. Thus, of 1074 DE mRNAs, 35.4% displayed corresponding increases or decreases in chromatin accessibility near their genetic loci (Fig. 6d). Chromatin accessibility was altered in the exon and promoter regions of 5 of 7 downregulated and 9 of 17 upregulated genes in the swMBC core transcriptional signature (Supplementary Fig. 5a–c). Gains in chromatin accessibility were associated with genes encoding proteins involved in MAPK, BCR, and Ca^2+^ signaling and leukocyte migration, validated by pathway analysis of the mRNA transcriptome (Fig. 5b). Losses in chromatin accessibility were associated with genes involved in Sphingolipid, MAPK, and stem-cell signaling, focal adhesion, and autophagy (Fig. 6e).

As the transcriptional regulation of human MBCs is poorly understood, we sought to determine whether MBCs DARs were associated with given *cis*-regulatory elements. Significant enrichment in DNA-binding factors that poise B cells for proliferation and plasma cell differentiation were found in DARs with increased accessibility in total MBCs, such as OCT2, IRF8, and bZIP motifs^43^. Conversely, enrichment in DNA-binding factors involved in B cell development and cellular quiescence, such as ERG, RHD, and KLF motifs^21,44^, were found in decreased accessible DARs of total MBCs (Fig. 6f). To further assess changes in TF activity, we probed for the enrichment of *cis*-regulatory elements in all accessible loci found in total MBCs over NBCs (Δ-MD score). NR1H4- and RXRA-binding motifs were enriched in the open chromatin regions of total MBCs while ERG1-, KLF-, and ZF-binding motifs were enriched in the open chromatin regions NBCs (Fig. 6g). These differences in motif accessibility were supported by MARINa-inferred changes in related TF activity. For example, BATF activity was inferred to be increased in swMBCs based on patterns of mRNA expression, and BATF-binding motifs were enriched in increased DARs in total MBCs (Figs. 5c and 6f, g).

Taiji was used to develop an integrative transcriptional regulatory network and predict TF activity based off a personalized PageRank algorithm. Consistent with our previous analyses, Taiji identified TFEC, RORα, and BATF as the TFs with the highest activity in MBCs, while KLF4, HEY2, and LEF1 were identified as the TFs with the highest activity in NBCs (Fig. 6h, i). Consistent with the notion that MBCs are poised to differentiate into plasma cells, PageRank inferred increased activity of PRDM1 and decreased activity of BCL6 in MBCs (Fig. 6h). Thus, the profile of chromatin accessibility contrasted human MBCs with NBCs and outlined signaling pathways and *cis*-regulatory elements relevant to MBCs.

### MicroRNAs in MBCs and identification of MIR181 as a major gene expression regulator

**Fig. 7:**
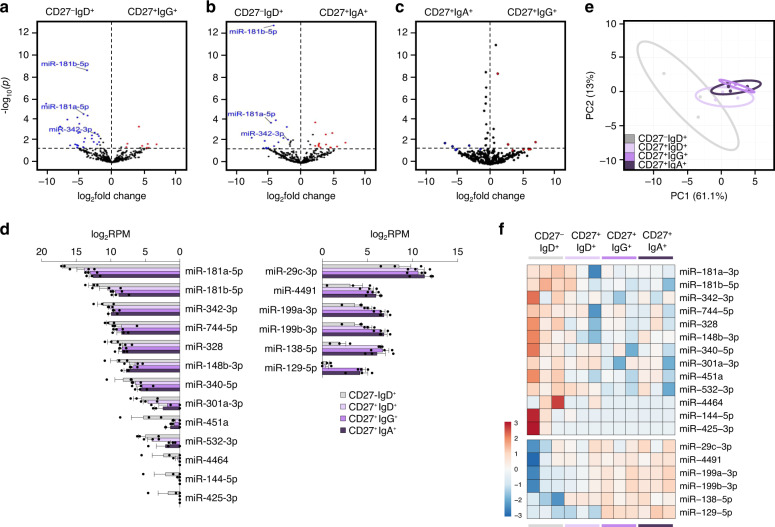
Altered expression of the miRNA profile in human swMBCs. **a**–**c** Global transcriptional differences of miRNAs in CD27^+^IgG^+^ vs. CD27^**–**^IgD^+^ B cells (**a**), CD27^+^IgA^+^ vs. CD27^**–**^IgD^+^ B cells (**b**), and CD27^+^IgG^+^ vs. CD27^+^IgA^+^ B cells (**c**) as depicted by volcano plot. All miRNAs annotated in human GENCODE v24 GRCh38 are shown, with each circle representing 1 miRNA. −Log_10_-transformed *p* is shown on the *y* axis (*p* < 0.05 indicated above dashed line). DE miRNAs at *p* < 0.05 are highlighted in red (upregulated) or blue (downregulated). DE miRNAs at *p* < 0.05 (with Δ abundance > 1000) shared by CD27^+^IgG^+^ and CD27^+^IgA^+^ are annotated. **d** Normalized (log_2_RPM) expression of the 19 DE miRNAs at *p* < 0.05 in swMBCs as compared to NBCs depicted by histogram for each B cell subset. Data are mean ± SEM of all three subjects. **e** Transcriptome clustering of the sorted subsets performed using the 19 DE miRNAs depicted by the PCA plot. Prediction ellipses define 95% confidence intervals. Each symbol represents an individually sorted subset (*n* = 3). **f** Relative expression profiles of swMBC core transcriptional signature miRNAs at *p* < 0.05 compared by heatmap, depicting relative transcri*p*tional changes across CD27^**–**^IgD^+^, CD27^+^IgD^+^, CD27^+^IgG^+^ and CD27^+^IgA^+^ B cell subsets in each subject (order; B, C, G). Data in **a**–**f** depict DE miRNAs as determined by edgeR. B cell subsets; CD27^–^IgD^+^ (gray), CD27^+^IgD^+^ (lavender), CD27^+^IgG^+^ (purple), and CD27^+^IgA^+^ (dark purple).

While chromatin accessibly is necessary for transcription, gene expression is subject to additional layers of regulation, mostly epigenetic, both at the DNA as well as post-transcriptional level. miRNAs are small ncRNAs, which target mRNA 3′ UTRs to post-transcriptionally repress gene expression^45^. As we have shown, miRNAs play a critical role in modulating B cell SHM, CSR, and plasma cell differentiation^28,29,45^. miRNAs role in MBCs is less understood. Our analysis of the miRNAome identified 19 DE miRNAs, 6 upregulated and 13 downregulated, in both IgG^+^ swMBCs and IgA^+^ swMBCs vs. NBCs at a significance of *p* < 0.05 (Fig. 7a, b). These 19 DE miRNAs “canceled out” when IgG^+^ swMBCs were compared with IgA^+^ swMBCs (Fig. 7c), being expressed at similar levels in IgG^+^swMBCs and IgA^+^ swMBCs (Fig. 7d and Supplementary Fig. 3c). Although also expressed in some cases in unswMBCs, they overall discriminated MBCs from their NBC counterparts (Fig. 7e, f). Among the 13 downregulated miRNAs in swMBCs, MIR181a and MIR181b, both MIR181 family members, were decreased by more than 60,000 and 3000 transcripts (Fig. 7d). MIR181a and MIR181b transcript downregulation were concomitant with decreased chromatin accessibility at these miRNA’s host–gene (HG) promoters (Supplementary Fig. 6a).

**Fig. 8:**
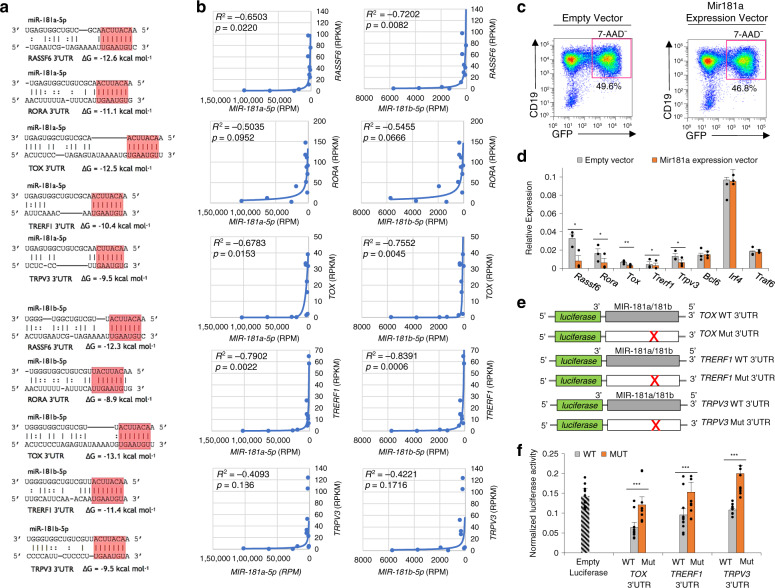
MIR181 as a key regulator of human swMBC-specific gene expression. **a** Schematic of 3′UTRs of *RASSF6, ROR*α*, TOX, TRERF1, TRPV3* mRNAs, and their targeting by MIR181a-5p and MIR181b-5p. Canonical target site base-pairing with miRNA seed sequences are highlighted in red. Hybridization is depicted as the most thermodynamically favorable molecular interaction between the two RNA species, considering RNA secondary structure. Gibbs free energy (Δ*G*) of nucleic acid folding and hybridization calculated by Mfold. Watson–Crick base-pairing (“|”); wobble base-pairing (“:”). Dashes indicate looping in the secondary RNA structure. **b** Spearman rank correlations of miRNA expression (RPM) and target mRNA expression (RPKM) calculated across all sorted subsets (*n* = 12). **c** FACs sorting of MDH1-PGK-GFP (Empty Vector) or MDH1-miR-181a-1-PGK-GFP (miR181 Expression Vector) retrovirally transduced (7AAD^-^CD19^+^GFP^+^) B cells. **d** Target gene expression (qPCR) in sorted (7AAD^−^CD19^+^GFP^+^) B cells transduced by MDH1-PGK-GFP (gray) or MDH1-miR-181a-1-PGK-GFP (orange) retrovirus (three independent experiments yielding similar results). *p < 0.05, **p < 0.01, ***p < 0.001, ns not significant (paired two-sided *t-*test). Data are mean ± SEM of three independent experiments. **e** Schematic diagram of the wildtype (WT) 3′UTRs of *TOX*, *TRERF1,* and *TRPV3* mRNAs and their mutant (Mut) counterparts cloned into the psiCHECK-2 reporter vector. Point-mutations in putative miRNA target sites are depicted by “X”. **f** Luciferase activity in human CL-01 cells transfected with constructs containing wildtype (gray) or mutated (orange) *TOX*, *TRERF1*, or *TRPV3* 3′UTRs (cloned into psiCHECK-2 luciferase reporter constructs) after 24 h stimulation with CD154 plus IL-4 (*n* = 10). Transfection efficiency was controlled for by normalization to *Renilla reniformis* signal encoded on the same psiCHECK-2 vector. *p < 0.05, **p < 0.01, ***p < 0.001, ns not significant (paired two-sided *t-*test). Data are mean ± SEM of three independent experiments.

Downregulation of MIR181a and MIR181b was expected to relieve the silencing of MIR181 targeted mRNAs. Indeed, 5 of the 17 significantly upregulated mRNAs in swMBC core transcriptional signature, *RASSF6*, *TOX*, *TRERF1*, *ROR*α, and *TRPV3*, were predicted targets of MIR181a and MIR181b, exhibiting 3’UTR sequences highly complementary with MIR181seed regions and favorable thermodynamics (Fig. 8a)—overall mRNA profile at *p*_adj_ < 0.05 showing 50 of the 462 mRNAs (10.8%) upregulated in both IgG^+^swMBCs and IgA^+^swMBCs to be predicted targets of MIR181 (Supplementary Fig. 6b). A significant inverse correlation between MIR181a/MIR181b and their 5 mRNA targets indicated the epigenetic release of these select mRNA transcripts in swMBCs (Fig. 8b). Indeed, enforced expression of Mir181a in retroviral-transduced mouse B cells significantly reduced the expression of these five genes by 32.3–72.4% (Fig. 8c, d and Supplementary Fig. 6c). Further, luciferase reporter assays involving (CD154 and IL-4) induced human CL-01 B cells expressing MIR181 and transfected with wildtype (WT) or mutant (Mut) reporter constructs confirmed *TOX*, *TRERF1,* and *TRPV3* to be direct and specific targets of MIR181 (Fig. 8e, f)—*RASSF6* was shown to be a direct target of MIR181 family members in the context of gastric cancer^46^; *ROR*α was not tested because of the length and complexity of its 3′UTR, which, however, contains three MIR181 target sites. Thus, MIR181 releases select swMBC gene expression from epigenetic regulation.

### Potential regulatory capacities of lncRNAs in human swMBCs

**Fig. 9:**
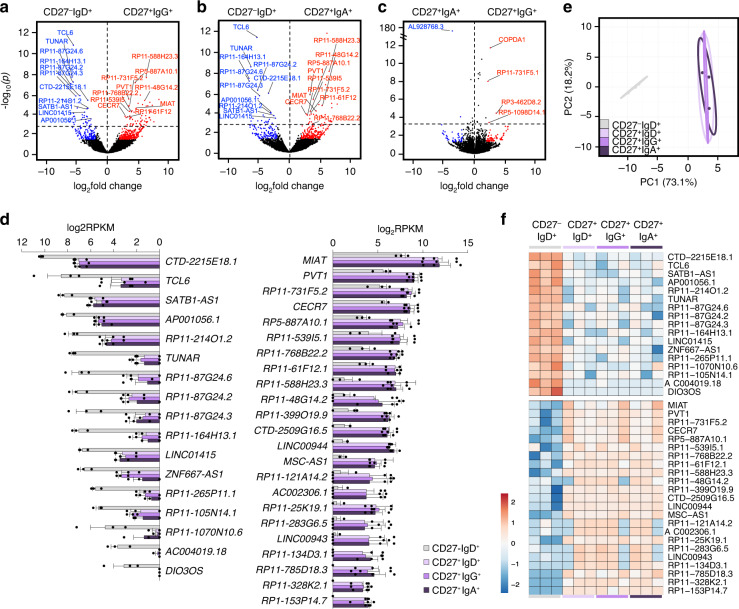
lncRNA expression profile in human MBCs and NBCs. **a–c** Global transcriptional differences of lncRNAs in CD27^+^IgG^+^ vs. CD27^–^IgD^+^ B cells (**a**), CD27^+^IgA^+^ vs. CD27^–^IgD^+^ B cells (**b**), as well as CD27^+^IgG^+^ vs. CD27^+^IgA^+^ B cells (**c**), as depicted by volcano plots. All lncRNAs annotated in human GENCODE v24 GRCh38 are shown, each circle representing 1 lncRNA. −Log_10_-transformed *p* is shown on the *y* axis (*p* < 0.001 indicated above dashed line). DE lncRNAs at *p* < 0.05 are highlighted in red (upregulated) or in blue (downregulated). DE lncRNAs at *p* < 0.001 (with Δ abundance > 100) common to both CD27^+^IgG^+^ and CD27^+^IgA^+^ are annotated. **d** Normalized (log_2_RPKM) expression of the top 40 DE lncRNAs at *p* < 0.001 in swMBCs as compared to NBCs depicted by histogram for each B cell subset. Data are mean ± SEM of all three subjects. **e** Transcriptome clustering of the sorted subsets performed using the 40 DE lncRNAs depicted by the PCA plot. Prediction ellipses define 95% confidence intervals. Each symbol represents an individually sorted subset (*n* = 3). **f** Relative expression profiles of swMBC core transcriptional signature lncRNAs at *p* < 0.001 compared by heatmap, depicting relative transcriptional changes across CD27^–^IgD^+^, CD27^+^IgD^+^, CD27^+^IgG^+^, and CD27^+^IgA^+^ B cell subsets in each subject (order; B, C, G). Data in **a**–**f** depict DE lncRNAs as determined by edgeR. B cell subsets; CD27^–^IgD^+^ (gray), CD27^+^IgD^+^ (lavender), CD27^+^IgG^+^ (purple), and CD27^+^IgA^+^ (dark purple).

lncRNAs are greater than 200 nucleotides in length and can interact with DNA, RNA, and proteins, thereby mediating a layer of epigenetic regulation to positively or negatively modulate gene expression^47^. lncRNAs have been implicated in key processes of T cells and innate lymphocytes^47^. While profiled in B cells^48^, their functional significance in human MBCs has not been explored. A comprehensive analysis by pairwise comparisons at *p* < 0.001 of MBC lncRNA landscape identified the same 23 upregulated and 17 downregulated DE lncRNAs in IgG^+^ swMBCs and IgA^+^ swMBCs as compared to NBCs—among these 40 DE lncRNAs, 21 had a change in abundance of more than 100 transcripts (Fig. 9a, b). All 40 lncRNAs “canceled out” when comparing IgG^+^ swMBCs vs. IgA^+^ swMBCs (Fig. 9c). Such 40 lncRNAs were indeed expressed at similar levels in IgG^+^ swMBCs and IgA^+^ swMBCs as well as in unswMBCs, thereby discriminating total MBCs from NBCs (Fig. 9d–f and Supplementary Fig. 3d).

**Fig. 10:**
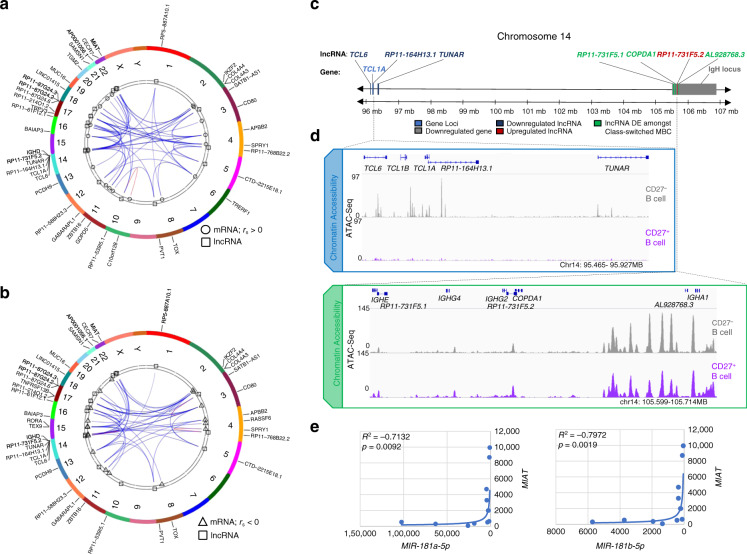
Characterization of the human MBC lncRNA profile. **a**, **b** lncRNA-to-mRNA co-expression correlations depicted as Circos plots with human chromosomal ideograms (various colors). lncRNA-to-mRNA correlations showing 21 DE lncRNAs (Δ abundance>100; depicted as squares) in swMBCs and the top three positively (**a** circles, *r*_s_ > 0) correlated mRNAs and top three negatively (**b** triangles, *r*_s_ < 0) correlated mRNAs for each lncRNA with *p* < 0.05 (Pearson correlation). **c** Schematic of the terminal end of chromosome 14 (q32.2–q32.3), depicted with DE mRNAs, lncRNAs, and critical B cell gene loci annotated. DE mRNAs and DE lncRNAs in swMBCs, as well as differences in genetic loci between CD27^+^IgG^+^ swMBCs, CD27^+^IgA^+^ swMBCs, and CD27^–^IgD^+^ NBCs are depicted. Downregulated lncRNAs (dark blue), upregulated lncRNAs (dark red), DE lncRNAs between swMBCs (green), downregulated mRNA (light blue), and the *IgH* locus (gray). **d** Chromatin accessibility upstream and in the human *IgH* loci is displayed by IGV gene track. Coverage includes gene and lncRNA introns, exons, promoter regions, and potential enhancer regions. ATAC-Seq signal is normalized for the window of interest, with NBCs depicted in gray and total MBCs in purple. **e** Correlations between expressed miRNAs and MIAT as calculated across all sorted subsets (*n* = 12) by Spearman’s rank correlation.

lncRNAs can modulate transcription of specific gene loci, generally *in cis* and nearby, including protein-coding loci (mRNA) and HGs of ncRNAs, such as miRNAs^47^. lncRNA functions have been inferred from lncRNA/mRNA co-expression analyses paired with functional and spatial enrichment. By integrated co-expression analysis, we identified significant positive and negative correlations between DE lncRNAs and DE mRNAs in swMBCs as compared to NBCs (Fig. 10a, b). One cluster of positively correlated transcripts found at the telomeric end of chromosome 14, upstream of the *IgH* locus contained 3 downregulated lncRNAs, *TCL6*, *RP11-164H13.1,* and *TUNAR*, together with 1 downregulated mRNA, *TCL1A* (Fig. 10c). The decreased expression of such lncRNAs and mRNA reflected the decreased chromatin accessibility at their respective loci (Fig. 10d). Interestingly, lncRNA *AL928768.3* found to be expressed at 10.8-fold higher levels in IgA^+^MBCs than IgG^+^ MBCs is located 2.5 kB downstream of the *IGHA1* locus. lncRNAs *COPDA1* and *RP11-731F5.1* were found to be expressed at 6.0 and 4.6-fold higher levels in IgG^+^ MBCs than IgA^+^ MBCs and are located in promoters of *IGHG2* and *IGHE* loci, respectively (Fig. 10c, d). *MIAT*, a molecular sponge for MIR181b^49^, was consistently upregulated in swMBCs and unswMBCs of all three subjects analyzed and displayed significant negative correlations with MIR181a and MIR181b expression (Fig. 10e).

To outline potential lncRNA regulatory functions, *trans* and *cis* co-expression correlations of lncRNAs with mRNAs or miRNAs were analyzed in swMBC, unswMBCs, and NBCs (Supplementary Fig. 7a–d). *Trans* lncRNA-mRNA correlations trended positive (*r*_s_ > 0.7, 3.29%) than negative (*r*_s_ < −0.7, 2.60%), as did *cis* lncRNA-mRNA correlations (*r*_s_ > 0.7, 3.97%; *r*_s_ < −0.7, 3.05%) (Supplementary Table 3). lncRNAs with strong positive or negative *cis* correlations (*r*_s_ > 0.65 or *r*_s_ < −0.65) with mRNAs genes revealed enrichment in lipid metabolic genes and GPCR signaling (Supplementary Fig. 7e, f). Thus, a lncRNA expression profile is characteristic of all CD27^+^ MBCs as compared to NBC, with select lncRNAs proximal to the *IgH* locus displaying potential regulatory activity or acting as miRNA sponges and overall co-expression patterns of different RNA species indicating enrichment in distinct signaling pathways.

## Discussion

mRNA, miRNA, and lncRNA transcriptomes revealed a common and unique transcriptional profile of human IgG^+^swMBCs and IgA^+^swMBCs, distinguishing them from NBCs. At the highest level of significance, swMBCs differed from NBCs in the expression of 24 genes, 17 increased and 7 decreased, with unswMBCs displaying a transcriptional signature of transition. The swMBC transcriptome was enriched in distinct MAPK, migratory and cytokine signaling pathways, likely influencing survival, homing and cell-to-cell interactions. In all (CD27^+^) MBCs, the chromatin landscape reflected changes in gene transcription and outlined differentially accessible *cis*-regulatory elements, potentially influencing cell gene expression and functions. As compared to NBCs, swMBCs non-coding transcriptome revealed a characteristic downregulation of MIR181a/MIR181b, pointing to this miRNA as a key modulator of swMBC gene expression. Also, lncRNA *MIAT*, a molecular sponge of MIR181^49^, was significantly increased in such swMBCs. *MIAT* likely released target genes from MIR181-mediated silencing, in concert with decreased accessibility of MIR181 HG promoters. Thus, when compared to NBCs, swMBCs exhibit a distinct mRNA, miRNA, and lncRNA transcriptome, dynamically interacting with a changing chromatin landscape to shape the identity and functions of these B cells.

CD27^–^IgD^+^, CD27^+^IgD^+^, CD27^+^IgG^+^, and CD27^+^IgA^+^ B cells displayed a pattern of *IgV*_H_ gene expression conserved across NBCs, unswMBCs, and swMBCs within each subject and reflecting the genomic representation of the seven V_H_ gene families. This was associated with a conserved distribution of CDR3 lengths in recombined V_H_DJ_H_-C_H_ transcripts, indicating an unbiased clonal differentiation of MBCs from their NBC progenitors. This was further supported by the conservation of Ig J_H_, Vκ, Jκ, Vλ and Jλ gene expression across NBCs, unswMBCs, and swMBCs, vastly overlapping across the subjects studied. Reflecting on a different degree of antigen experience, the mutational load in recombined transcripts was the greatest in swMBCs, intermediate in unswMBCs, and negligible in NBCs.

Our findings outlined a distinct gene expression profile characteristic of human swMBCs, regardless of their effector Ig class, be it IgG^+^ or IgA^+,^ and possibly IgE^+^. They extend previous studies, which addressed select human MBCs, as conditioned by different antigens, microenvironments, and/or biased genetic programs^41^, and identified transcriptional programs not associated with MBCs of specific effector Ig classes^25,50^. In our seven subjects, NBCs accounted for two-thirds of the B cell repertoire, with unswMBCs and (IgG^+^ and IgA^+^) swMBCs making up for about one-tenth each. The 17 upregulated and seven downregulated mRNA transcripts distinguishing swMBCs from NBCs “canceled out” when comparing IgG^+^ MBCs with IgA^+^ MBCs, leaving the reciprocal *IgH* expression of the respective *IgCγ* and *IgCα* transcripts as the distinguishing difference. Expression of *IgCε* transcripts in IgG^+^ MBCs, but not in IgA^+^MBCs likely reflected the transition to IgE through sequential CSR from IgM to IgG1 and to IgE^42^.

The phenotype, transcriptome, functional cellular pathways, and gene loci accessibility yielded unifying information on MBCs of different isotypes, as different from NBCs in the same subjects. This was unexpected and per se remarkable, as we found a consistent homogeneity and concordance in the mRNAome, miRNAome, lncRNAome, chromatin accessibility, and inferred pathways of cell functions within MBCs—whether considered as CD27^+^IgG^+^ swMBCs and CD27^+^IgA^+^ swMBCs or total CD27^+^ B cells vs. NBCs, using two different B cell isolation approaches (isotype-specific MBCs and total MBCs) and despite the heterogeneous origin of the B cell samples: seven subjects of different age, sex, and race—a cohort quite different from any cohort of inbred mouse strains. Single-cell RNA-Seq may resolve further DE genes beyond this core transcriptional signature.

The identity and role of unswMBCs (CD27^+^IgD^+^) are a matter of debate. These B cells have been suggested to act as a reservoir of elements ready to supplement a recall antibody response, particularly in parasitic and bacterial infections^24,51–53^. Our findings suggest a transitional identity for human unswMBCs. These B cells expressed IgV_H_, J_H_, Vκ, Jκ, Vλ, and Jλ genes as well as a V_H_DJ_H_-C_H_ CDR3 length distribution similar to those of NBCs and swMBCs, and carried a moderate load of somatic point-mutations, intermediate between NBCs and swMBCs, consistent with unswMBCs emerging from NBCs and being potential precursors of swMBCs. This transitional identity is further emphasized by: (i) the intermediate expression of the 24 genes distinguishing swMBCs from NBCs; (ii) the unswMBCs hierarchical clustering pattern; (iii) the consistent downregulation of MIR181a and MIR181b, which is characteristic of swMBCs; and (iv), finally, the differential lncRNA profile nearly identical to that of swMBCs.

Of the 17 mRNAs upregulated in swMBCs at the highest level of significance, 6 transcripts, *AIM2, CD80, RASSF6, ROR*α*, TACI,* and *TOX*, were reported to be upregulated in human CD27^+^B cells, mouse IgA^+^ MBCs or NP-specific GC-derived memory precursors, thereby emphasizing the importance of such genes also in human IgG^+^ MBCs and IgA^+^ MBCs^17,20,50,54–57^. Baff-R signaling would be important in the survival of mouse MBCs^58^. *BAFFR* was expressed in human NBCs and swMBC, while *TACI*, another BAFF receptor, was upregulated in swMBCs, thereby suggesting similar signaling requirements for mouse and human MBC survival. *BAIAP3*, *TGM2,* and *TRPV3* encode proteins critical to ion channel Ca^2+^ flux, Ca^2+^ signal transduction, or Ca^2+^-dependent activity, likely playing a role in MBC reactivation^59^. *TOX*, *TRERF1,* and *ROR*α, encode for TOX, a high-mobility group (HMG) box protein, which binds DNA architectural motifs and is critical in immune cell development^60^, TRERF1, which interacts with p300 and SF-1 to regulate P450scc gene expression, thereby impacting the cell cycle^61^, and RORα.

*ROR*α was highly expressed in IgG^+^ MBCs and IgA^+^ MBCs, but not in NBCs, suggesting a central role for this TF in the identity of human swMBCs. RORα is a sequence-specific ligand-dependent TF implicated in mouse ILC2 and Th17 cell differentiation as well as mouse IgA^+^ MBC maintenance^20,62^. RORα was found to be central to the transcriptional network of MARINa-inferred TFs, nucleus-localized swMBC signature genes, and IPA-identified coactivators and corepressors. This indicates RORα’s importance in MBC identity and/or functions, as further supported by the integrative Taiji analysis. RORα’s high expression in peripheral blood and tonsil swMBCs further suggested an important role of this TF in MBCs maintenance. *MUC16*, which encodes a 22,000 amino acid membrane-associated adhesion molecule, was significantly upregulated in swMBCs. Through its binding partners galectin-1, galectin-3, and L-selectin, abundantly expressed by CD169^+^ macrophages^63^, Mucin-16 would stabilize cell-to-cell contact between MBCs and CD169^+^ subcapsular sinus macrophages to promote antigen subsequent B cell reactivation^64^. Mesothelin, another Mucin-16 binding partner, potentially home MBCs to lungs, consistent with reports of influenza-specific lung-resident MBCs in the mouse^65,66^. Thus, alongside changes in leukocyte migratory signaling and positional cues, the upregulation of *MUC16* could suggest unique trafficking patterns that promote antigen surveillance by MBCs in the respiratory tract.

The downregulation at the highest level of significance of seven mRNAs in swMBCs suggested these genes are not essential for differentiation or maintenance of these B cells. Such genes included *ZBTB16*^21^ (ZBTB16’s activity was inferred by MARINa to be significantly decreased in swMBCs as compared to NBCs) as well as six other genes. Of these, *IKZF2* codes for a key TF in early B cell development^67^ and double negative self-reactive T cells^68^, while TCL1A, an AKT signal transduction molecule, is downregulated in the transition from NBCs to GC B cells^69^. PCDH9, is a Ca^2+^ dependent cell-adhesion protein^70^ and SPRY1 a negative regulator of cell proliferation, migration, and promoter of apoptosis^71^.

At a lower level of significance (*p*_adj_ < 0.05), swMBCs’ gene expression profile comprised transcripts that were previously reported, including upregulated *CD86*, *CXCR3*, *EBI2*, *FAS*, and *ZBTB32*, and downregulated *KLF4* and *KLF9*^21,50,63,72^. IgG^+^ swMBCs and IgA^+^ swMBCs displayed decreased expression of *BACH2*, a gene that skews mouse MBCs toward plasma cell differentiation, upon reactivation^18^. In unswMBC and swMBCs, the expression of *IL-9* and *IL-9R* was virtually absent, suggesting that IL-9 signaling, which is putatively important for mouse MBC generation^17^, is dispensable for human MBC maintenance and, perhaps, generation. While *IL-2RG* was abundantly expressed in all four B cell subsets, cytokine receptor subunits that pair with the common-γ chain were differentially expressed in NBCs and swMBCs. Accordingly, human swMBCs downregulate the expression of *IL-4R*, *IL-21R,* and *IFN*γ*R1*, whose signaling contributes to T-bet expression^73^. In the healthy humans we analyzed, *TBX21* expression was absent in swMBCs and unswMBCs, consistent with the segregation of T-bet^+^ “atypical” MBCs among double negative (CD27^–^IgD^–^) B cells^74^.

As assessed by ATAC sequencing, the genes whose transcription was significantly upregulated in swMBCs were highly accessible, emphasizing the importance of such genes to the identity of swMBCs. While chromatin accessibly is necessary for transcription, it is not sufficient per se for gene expression. Gene transcription is regulated by DNA methylation, histone post-translational modifications and activators, and repressors^34,35,75^. Additional modulation of gene expression is mediated by epigenetic factors, mainly, non-coding RNAs, such as miRNAs and lncRNAs, at the transcriptional and post-transcriptional level. 13 of 17 gene loci, including *RASSF6*, *TRPV3*, *TOX,* and *ROR*α, were significantly accessible in NBCs, despite negligible levels of the respective transcripts, suggesting mechanisms of post-transcriptional regulation, possibly mediated by ncRNAs.

In this context, we showed MIR181a and MIR181b to be key regulatory ncRNAs, likely acting as epigenetic repressors of MBC-specific gene expression. To date, there has been no investigation addressing the interplay of miRNAs and mRNAs in human MBCs. Our findings showed a great reduction of MIR181a and MIR181b expression in the transition from NBCs to unswMBCs and swMBCs, a transition that would occur after the GC stage^37,76^. Chromatin accessibility in promoter regions of MIR181 HGs on chromosomes 1 and 9 was reduced in total MBCs, suggesting that decreased MIR181a/MIR181b expression was mediated in part, by chromatin remodeling. Integrated analysis of the miRNA and mRNA data sets predicted MIR181 to further target 11% of the greater upregulated MBC genes (50/462 genes) and 5 of the 17 DE mRNAs upregulated in swMBC. MIR181’s role in regulating such genes was supported by MIR181 complementarity to target gene 3′UTRs and strengthened through functional evidence. Enforced expression of Mir181 in B cells significantly reduced *Rassf6*, *Tox*, *Trerf1*, *Ror*α, and *Trpv3* expression, while luciferase reporter assays confirmed *RASSF6*, *TOX*, *TRERF1,* and *TRPV3* as direct and specific targets of MIR181, thereby identifying a novel epigenetic mechanism centered on MIR181 and impacting key gene expression in swMBCs.

In human tonsils, lncRNAs display a dynamic relationship between enhancer lncRNAs and genetic elements of GC B cells^48^. Our findings showed that human circulating IgG^+^ swMBCs and IgA^+^ swMBCs as well as unswMBCs displayed nearly identical lncRNA profiles, that discriminate them from their NBC progenitors. In swMBCs, distinct DE lncRNAs disproportionately clustered proximal to or within the *IgH* locus, a key element of BCR and antibody expression. Accordingly, DE lncRNAs in IgG^+^ MBCs vs. IgA^+^ MBCs were associated with either *IgCγ*2, *IgCε*, or *IgCα*1 gene exons, thereby indicating a role for such lncRNAs in the regulation of switched BCR expression. Integration of DE mRNAs with DE miRNAs, as complemented by functional assays, identified MIR181 downregulation as a central mechanism of swMBC-specific gene expression. *MIAT*, a sponge of MIR181^49^, was upregulated in swMBCs and inversely correlated with MIR181a and MIR181b expression, suggesting that *MIAT* provides a regulatory mechanism that, along with chromatin accessibility, contributes to reduced MIR181 expression and promotes swMBC-specific gene expression.

Our integrative analysis of transcriptional profiles and chromatin accessibility revealed a dynamic and synergistic epigenetic landscape distinguishing MBCs from NBCs, and suggested that swMBCs emerge stochastically from the NBC pool, not from select elements or subsets of NBCs. Overall, our findings point to a core transcriptional signature, which is characteristic of and shared by swMBCs, regardless of BCR isotype, be it IgG or IgA, and distinct from that of NBCs. Beyond this core transcriptional signature, single-cell RNA-Seq may further tease apart the heterogeneity of human swMBCs, perhaps identifying discrete elements within this memory compartment^77^. UnswMBCs may act as intermediate elements in the transition of NBCs to swMBCs, which trend closer to swMBCs, consistent with their CD27 expression, light mutational load, Euclidean clustering, and non-coding transcriptional profile. Further, our findings, which stem from the integration of chromatin remodeling, *cis*-regulatory elements, and distinct expression profiles of mRNAs, miRNAs, and lncRNAs, provide evidence of a key regulatory role for the non-coding transcriptome, particularly MIR181 and *MIAT*, in the identity, maintenance and, likely, generation of human MBCs. Finally, by providing new insights into the transcriptional and chromatin landscape of MBCs, they open new avenues of investigation on the generation, key drivers, such as RORα, TRERF1, and TOX, identity, and role of MBCs as critical cellular elements in human health and disease.

## Methods

### Human B cells isolation

Buffy coats were obtained from South Texas Blood and Tissue Center, San Antonio, TX. Samples were obtained from seven healthy and non-immuno-compromised human subjects of different ages, 20–39, four males, three females of a different races, and ethnic backgrounds (Subjects A–G) (Supplementary Table 1)^78^. All experiments involving human samples were approved by the Institutional Review Board (IRB) of UTHSCSA and written informed consent was obtained. PBMCs were isolated from a diluted buffy coat (1:2) using density gradient Histopaque (Sigma Aldrich) in SepMate 50 ml tubes, according to the manufacturer’s instructions^79^. PBMCs were separated from the RBCs and subjected to two washes with FCS-RPMI (RPMI 1640, 10% fetal bovine serum, 1% antibiotics) and spun at 0.3 g for 8 min. Residual RBCs were lysed using ACK lysing buffer (ThermoFisher Scientific) and quenched using full media. Cell counting was performed on a Bright-Line™ hemocytometer (Hausser Scientific) utilizing a Nikon Labophot-2 research microscope. Approximately 5 × 10^8^ PBMCs were obtained per subject. PBMCs were resuspended to 50 × 10^6^ cells/ml.

B cells (total) were isolated by negative selection from PBMCs (subjects B, C, G) using the EasySep Human Total B Cell Isolation Kit (STEMCELL Technologies). This kit utilizes anti-CD4, CD8, CD14, CD16, CD25, CD36, CD56, CD61, CD66b, CD123, and glycophorin A mAbs conjugated to anti-dextran Abs, which in conjunction with dextran-coated magnetic beads allow for depletion of all cell types other than B-cells. Approximately 5 × 10^7^ B cells were obtained from each subject, validated for purity (>99%) by flow cytometry by anti-CD19 mAb and used for cell sorting using a BD LSRII flow cytometer (BD Biosciences) or BD FACSCelesta flow cytometer (BD Biosciences) with FACSDiva software (BD Biosciences). Seventy-five percent of these B cells were stained with FITC-anti-human-IgG mAb (clone G18-145; BD Pharmingen), APC-anti-human-IgA mAb (clone IS11-8E10; Miltenyi Biotec), and PE-anti-human-CD27 mAb (clone M-T271; Biolegend) for isolation of swMBCs. CD27^+^IgG^+^ B cells and CD27^+^IgA^+^ B cells were sorted into separate 1.2 ml Eppendorf tubes containing 0.5 mL of HBSS (Hank’s balanced salt solution) buffer containing 0.1% bovine serum albumin (BSA). The remaining 25% of B cells were stained with anti-IgD BV421 mAb (clone IA6-2; Biolegend) and anti-CD27 PE mAb (clone M-T271; Biolegend), and CD27^–^IgD^+^ B cells and CD27^+^IgD^+^ B cells were sorted in 0.5 ml of HBSS + 0.1% BSA buffer. All sorted B cells were subjected to phenotypic analysis for confirmation of their identity and purity.

CD27^−^ and CD27^+^ B cells were directly isolated from PBMCs (Subjects A, D, E, F) by immunomagnetic selection using the EasySep Human Memory B Cell Isolation Kit (STEMCELL Technologies). This is based on a proprietary two-step method using EasySep Releasable RapidSpheres to positively select CD27^+^ cells, remove magnetic particles, and then deplete non-B cells using EasySep Dextran RapidSpheres with antibody complexes. This procedure also allows for the depletion of non-B cells in the CD27^–^ fraction in order to isolate naive B cells in parallel.

In all seven cases, B cells were subjected to pre-and-post isolation phenotypic analysis using the following surface markers and fluorophores: PEcy7-anti-human-CD19 mAb (clone H1B19; Biolegend; 1:100), PE-anti-human-CD27 mAb (clone M-T271; Biolegend; 1:100), BV421-anti-human-IgD mAb (clone IA6-2; Biolegend; 1:100), FITC-anti-human-IgG mAb (clone G18-145; BD Pharmingen; 1:100), APC-anti-human-IgA mAb (clone IS11-8E10; Miltenyi Biotec; 1:100), and APC-Cy7-anti-IgM mAb (clone MHM-88, Biolegend; 1:100). Data were analyzed using FlowJo software version 10.6.2 (FlowJo LLC)^78^.

### Flow cytometry

PBMCs or B cells were stained with fluorochrome-conjugated mAbs in Hank’s Buffered Salt Solution plus 0.1% BSA (BSA-HBSS) for 20 min. Total PBMCs were stained with FITC-anti-human-IgG mAb (clone G18-145; BD Pharmingen; 1:100), APC-anti-human-IgA mAb (clone IS11-8E10; Miltenyi Biotec; 1:100) and APC-Cyanine7-anti-human-CD27 mAb (clone M-T271; Biolegend; 1:100), BV421-anti-human-IgD mAb (clone IA6-2; Biolegend; 1:100), PE-Cyanine7-anti-human-CD19 (clone HIB19; Tonbo Biosciences; 1:100). Single cell suspension of tonsil cells were stained with APC-Cyanine7-anti-human-CD27 mAb (clone M-T271; Biolegend; 1:100), BV421-anti-human-IgD mAb (clone IA6-2; Biolegend; 1:100), PE-Cyanine7-anti-human-CD19 (clone HIB19; Tonbo Biosciences; 1:100), BV510-anti-human-CD138 mAb (clone MI15 Biolegend; 1:100), BV650-anti-human-CD38 mAb (clone HB-7; Biolegend; 1:100)^30,32,78^. For intracellular staining, cells were surface stained followed by incubation with the BD Cytofix/Cytoperm buffer at 4 °C for 20 min. After washing twice with the BD Perm/Wash buffer, cells stained with PE-anti-human-RORα mAb (Clone 784652; R&D Systems; 1:50) overnight at 4 °C. Cells were then were resuspended in HBSS with 1% BSA for flow cytometry. In all flow cytometry analyses, cells were appropriately gated on forward and side scattering to exclude dead cells and debris^28^. RORα expression in CD27^-^IgD^+^, CD27^+^IgD^+^, CD27^+^IgG^+^ and CD27^+^IgA^+^ B cells from peripheral blood of the 7 healthy human subjects was analyzed by intracellular staining followed flow cytometry. RORα expression in B cell fractions from tonsils of three additional human subjects were stained intracellularly and analyzed by flow cytometry. Fractions analyzed comprise NBCs (CD19^+^CD38^−^CD27^−^IgD^+^CD138^−^), GC B cells (CD19^+^CD38^lo^CD27^+/−^IgD^+/−^CD138^−^), swMBCs (CD19^+^CD38^−^CD27^+^IgD^−^CD138^−^), DN2 MBCs (CD19^+^CD38^−^CD27^−^IgD^−^CD138^−^), plasmablasts and plasma cells (CD19^+/^^−^CD38^hi^CD27^+^IgD^−^CD138^+/^^−^). Samples were run using a BD LSRII flow cytometer (BD Biosciences) or BD FACSCelesta flow cytometer (BD Biosciences) with FACSDiva software (BD Biosciences). Seven‐parameter files were imported into FlowJo software version 10.6.2 and biexponentially transformed prior to *t*-SNE analysis. *t*-SNE was then run on concatenated down-sampled files (100,000 cells/sample). *t*-SNE maps were generated by plotting *t*-SNE dimension 1 and dimension 2, and the gated subsets overlaid manually based on markers of interest on the dot-plot, to show the expression of these markers on different tonsil subtypes.

### mRNA, miRNA, and lncRNA sequencing

RNA was isolated from cells using the Directzol RNA Microprep Kit (Zymogen Research) (based on cell number), according to the manufacturer’s instructions and as previously described^30^. RNA integrity was verified using an Agilent Bioanalyzer 2100 (Agilent). Next-generation RNA-Seq for mRNA and non-coding RNA was performed by the Genome Sequencing Facility at the University of Texas Health Science Center San Antonio (UTHSCSA) Greehey Children’s Cancer Research Institute. High-quality RNA was processed using an Illumina TruSeq RNA sample prep kit v2 or TruSeq Small RNA Sample Prep Kit following the manufacturer’s instructions (Illumina). Clusters were generated using TruSeq Single-Read Cluster Gen. Kit v3-cBot-HS on an Illumina cBot Cluster Generation Station. After quality control procedures, individual mRNA-Seq or small RNA-Seq libraries were then pooled based on their respective 6-bp index portion of the TruSeq adapters and sequenced at 50 bp/sequence using an Illumina HiSeq 3000 sequencer. Resulting reads were checked by assurance (QA) pipeline and initial genome alignment (Alignment).

### IgV_H_, D, J_H_, and V_L_, J_L_ gene expression

To analyze expressed Ig V_H_, D, J_H_ and Vκ, Jκ, as well as Vλ, Jλ genes in CD27^–^IgD^+^, CD27^+^IgD^+^, CD27^+^IgG^+^, and CD27^+^IgA^+^ B cells, we collated the transcript read counts (RPKM) that aligned to the IgH and IgL chain genes to determined relative composition profiles for all 12 samples sorted. The human *IgH* locus comprises seven families with 36–49 functional coding V_H_ genes: V_H_1, 11 members; V_H_2, 4 members; V_H_3, 23 members; V_H_4, 7 members; V_H_5, 2 members; V_H_6, 1 member. The V_H_7 family consists of only one member, *IGHV*7-4-1, which is not always part of a haplotypic complement, i.e., is absent in some subjects. The human Igκ locus comprises 39 functional Vκ genes and 5 Jκ genes; the human Igλ locus comprises 30 functional Vλ genes segregated into 10 subgroups and 5 functional Jλ-Cλ clusters. IgH and IgL gene expression profiles were organized in stacked histograms to depict relative usage^39,80^ (The International ImMunoGeneTics Information System, http://www.imgt.org/). Stacked histograms were also used to depict Ig V_H_, D, J_H_ and Vκ, Jκ, as well as Vλ, Jλ gene segments genomic representation.

### IgH CDR3 and V_H_ somatic point-mutations

Recombined V_H_DJ_H_-C_H_ transcripts were amplified to determine CDR3 length and analyze somatic point-mutations in V_H_ segments^28,78^. RNA was extracted using the Directzol RNA Microprep Kit (Zymogen Research) from CD27^-^IgD^+^, CD27^+^IgD^+^, CD27^+^IgG^+^, and CD27^+^IgA^+^ B cells of subjects B, C, and G. cDNA was synthesized from RNA with the SuperScript III First-Strand Synthesis System (Invitrogen) using oligo-dT primer. Rearranged V_H_DJ_H_-C_H_ cDNA was amplified using forward (degenerate) primers for leader sequences of (V_H_1, V_H_2, V_H_3, V_H_4, V_H_5, V_H_6, V_H_7 family genes) in conjunction with reverse Cμ, Cδ, Cγ or Cα isotype-specific primer and Phusion DNA polymerase (New England BioLabs). Amplification conditions were 98 °C for 30 s, 58 °C for 30 s, and 72 °C for 1 min. The amplified library was tagged with Illumina clustering adapters with barcodes for sample multiplexing and enriched by PCR. High-throughput, 300 bp pair-ended sequencing was performed using the Illumina MiSeq system. Somatic point-mutations were identified by aligning V_H_ segments of recombined V_H_DJ_H_ sequences with their respective germline templates using IMGT/HighV-QUEST (The International ImMunoGeneTics Information System, http://www.imgt.org) and corrected for polymerase and sequencing error rates (0.008) to calculate the frequency of mutations (change/base)^81^.

### ATAC-seq

The Omni-ATAC adapted protocol was used for ATAC-Seq preparation^82^. This protocol minimizes mitochondrial DNA contamination, increases library complexity, and signal-to-noise ratio. Briefly, human MBCs or NBCs were isolated from PBMCs through immunomagnetic selection using the EasySep Human MBC isolation according to the manufacturer’s instructions (Stemcell EasySep). MBCs (30,000) or (30,000) NBCs were centrifuged at 500×*g* for 5 min at 4 °C, washed with 50 μl of cold 1× PBS, and centrifuged again at 500×*g* for 5 min at 4 °C. After discarding the supernatant, the pelleted cells were resuspended in 50 μl cold lysis buffer (10 mM Tris pH 7.4, 10 mM NaCl, 3 mM MgCl_2_, 0.1% NP-40, 0.1% Tween-20, 0.01% Digitonin), incubated on ice for 3 minutes, quenched with 500 μl cold Tween-only-buffer (10 mM Tris pH 7.4, 10 mM NaCl, 3 mM MgCl_2_, 0.1% Tween-20) and centrifuged at 500×*g* for 30 min at 4 °C. After removing the supernatant, nuclei were immersed in (30 μl) tagmentation reaction mixture (5 μl 5× insertion buffer, 2 μl Tn5-50 transposase, 8 μl sterile PBS, 10 μl nuclease-free H_2_O) at 37 °C for 45 min. Such mixture was then diluted in (another 30 μl) 2× nuclei lysis/deactivation buffer (300 mM NaCl, 100 mM EDTA, 0.6% SDS, 1.6 μg Proteinase-K) before incubation for 30 min at 40 °C. DNA was isolated using Zymo DNA Clean and Concentrator kit according to manufacturer’s instructions, PCR amplified using Illumina compatible index primers and libraries were normalized through qPCR analysis. DNA was enriched for fragments <1000 bp using Zymo Select-a-Size DNA Clean and Concentrator MagBead kit according to manufacturer’s instructions. Samples were quality checked for on an Agilent Bioanalyzer 2100, pooled at an equimolar ratio, and sequenced on a HiSeq 3000 using 50 bp single-end chemistry. Peak calling, gene annotation, and enrichment of TF motifs were performed using HOMER, with dependencies in Samtools and Bedtools. Statistically significant DARs were determined using DESeq2. Significant changes in TF activity were detected using DAStk and HOMER. Peak coverage and annotation were performed using HOMER^34,35,83^.

### Retroviral Mir181 construct and enforced expression

A mouse Mir181a retroviral expression vector was used for overexpression experiments. Briefly, a 270-bp miRNA gene segment containing the Mir181 miRNA hairpin was cloned from mouse chromosome 1 into MDH1-PGK-GFP expression vector. To generate the retrovirus, MDH1-PGK-GFP expression vector, encoding GFP, or MDH1-miR-181a-1-PGK-GFP expression vector, encoding GFP and miRNA-181a, together with the pCL-Eco retrovirus-packaging vector (Imgenex) were used to transfect HEK293T cells by a Ca^2+^ phosphate-mediated transfection procedure. Viral supernatants were harvested and used to transduce (by spinning at 400 g for 45 minutes followed by culture with LPS plus IL-4 for 72 hrs) LPS-preactivated (12 hr) C57BL/6 mouse spleen B cells, as we reported^32,84^. CD19^+^GFP^+^7-AAD^−^ transduced B cells were sorted by FAC^32^. Expression of *Ror*α*, Tox, Trerf1, Trpv3,* and *Rassf6* transcripts in B cells transduced with empty or Mir181-expression retroviral construct was analyzed by quantitative RT-PCR^78,85^.

### Quantitative RT-PCR (qRT-PCR) of mRNAs and miRNAs

For the quantification of mRNA transcripts, RNA was extracted. cDNA was synthesized from total RNA with the SuperScript III First-Strand Synthesis System (Invitrogen) using an oligo-dT primer. Transcript expression was measured by qRT-PCR with the appropriate primers using a QuantStudio 3 Real-Time PCR System (ThermoFisher) to measure SYBR Green (IQ SYBR Green Supermix, Bio-Rad Laboratories) incorporation with the following protocol: 95 °C for 15 s, 40 cycles of 94 °C for 10 s, 60 °C for 30 s, 72 °C for 30 s. Data acquisition was performed during the 72 °C extension step. Melting curve analysis was performed from 72 to 95 °C. For the quantification of mature miRNA transcripts, RNA was extracted from 0.2 to 5.0 × 10^6^ cells using miRNeasy^®^ Mini Kit (Qiagen) and then reverse-transcribed with miScript II RT Kit (Qiagen) using the miScript HiSpec buffer. A Bio-Rad MyiQ Real-Time PCR Detection System was used to measure SYBR Green (miScript SYBR Green PCR Kit; Qiagen) incorporation, according to manufacturer’s instructions. Mature miRNA forward primers were used at 250 nM in conjunction with the Qiagen miScript Universal Primer and normalized to the expression of small nuclear/nucleolar RNAs Rnu6/RNU61/2, Snord61/SNORD61, Snord68/SNORD68, and Snord70/SNORD70. The 2^−ΔΔC^_T_ method was used for data analysis of qRT-PCR experiments.

### Luciferase 3′ UTR reporter assays

The 3′UTRs of *TOX*, *TRERF1,* and *TRPV3* mRNAs were cloned into the psi-CHECK2 miRNA Expression Reporter Vector System (Promega) downstream of the Firefly luciferase^28,85^. *TOX*, *TRERF1,* and *TRPV3* 3′UTR mutant psiCHECK-2 constructs were generated through site-directed mutagenesis of MIR181 target site seed sequences. The constructs were verified by sequencing. The psiCHECK-2 reporter also contained a Firefly luciferase reporter, which allows for normalization of Renilla luciferase activity between samples. Constructs were used to transfect human CL-01 B cells by electroporation (500 V, 950 μF, ∞ Ω) with a Gene Pulser II (Bio-Rad). Transfected CL-01 B cells were then stimulated with CD154 (1 U/ml) and IL-4 (5 ng/ml) for 24 h. The ability of endogenous MIR181 to repress reporter activity was determined by Firefly luciferase activity and normalized to Renilla luciferase activity, according to the manufacturer’s instructions, using the Luc-Pair™ Duo-Luciferase HS Assay Kit (GeneCopoeia).

### Bioinformatics

After sequencing, demultiplexing with CASAVA was employed to generate a Fastq file for each sample. Initial data processing was performed by the CBBI (Computational Biology and Bioinformatics Initiative) at UTHSCSA. All sequencing reads were trimmed with Trim Galore, aligned with their reference genome (UCSC hg19) using HISAT2 default settings^86^. Bam files from the alignment were processed using HTSeq-count to obtain counts per gene in all samples^87^. RNA expression levels were determined using GENCODE annotation (GENCODE human v24). mRNA and lncRNA sequencing generated 12–21 million reads per sample, while smRNA sequencing generated 0.6–2.5 million reads per sample. Differential expression analysis was performed using the EdgeR package in R post-normalization^88^. mRNA/lncRNA was removed from downstream analysis if it did not break the threshold of at least 1 RPKM mapped reads across all sample libraries. For V(D)J analysis, the threshold was set >5 mapped reads across all sample libraries to exclude genes that are not productively expressed (i.e., non-recombined). DE mRNA between two groups was determined based on a Benjamini–Hochberg false discovery rate (FDR)-corrected threshold for statistical significance of *p*_adj_ < 0.05. of DE miRNA and DE lncRNA between two groups was defined based on a criterion of *p* < 0.05. The transcript read counts were transformed to log_2_RPKM (reads per kilobase per million reads) and used to generate heatmaps as well as PCA plots in Clustvis^89^. Volcano plots depicting log_2_-fold change and raw or adjusted *p*-values were generated in RCircos plot ideograms using the Circos package in R.

### Ingenuity pathway analysis (IPA) and gene functional classifications

Network analysis was performed using IPA (Qiagen) to investigate biological pathways associated with DE genes in the swMBC core transcriptional signature. The IPA database is the largest curated database of published findings on human biology from the public literature. Canonical pathway analysis categorizes function-specific genes present within networks and determines the significance of those genes through statistical over-representation analysis. Further, for pathways with enough information, the directionality of upregulated and downregulated genes can be used to inform the activation or inactivation of a pathway between two states (i.e., NBCs and MBCs), which is quantified independently in IPA as *Z*-score. Thus, in different cells or different cell states, pathways can be statistically significant as well as be significantly activated or statistically significant and be significantly inactivated. The aggregate mRNA profile was analyzed to identify statistically over-represented pathways based on FDR-adjusted *p* values < 0.05 and to infer pathway activation status based on *Z*-score. For the biological function classifications shown in Fig. 5c, DE genes were manually curated using multiple public databases that include gene ontology annotation, as well as published literature.

### MARINa

Transcriptional regulators that may underlie the differential expression profiles of human MBCs and NBCs were inferred using the MARINa algorithm^90,91^ based on the available human B cell interactome. The regulatory direction (positive or negative) for each transcriptional regulator-target gene pair was defined previously^90^ and developed into a regulon object for use in R with the VIPER package. Gene expression was ranked from most downregulated to most upregulated genes between NBCs and MBCs. MARINa quantifies differential activities of transcriptional regulators by measuring enrichment of predicted targets at the terminal ends of this differential gene signature. For any given regulator, enrichment of its positively regulated targets among upregulated genes as well as enrichment of its negatively regulated targets among downregulated genes suggested higher regulator activity in MBCs. The converse was true if positively regulated targets were enriched among downregulated genes and negatively regulated targets were enriched among upregulated genes, indicating lower regulator activity in MBCs. 1000 sample permutations were used to calculate a TFs enrichment score and statistical significance (*p* < 0.05), which was taken to suggest that a given TF was a potential driver of differences between NBCs and MBCs.

MARINa is the only cell regulatory network model that is solely based on data sets from human B cells (performing better than standard Bayesian networks), therefore making it a good fit for our studies. The MARINa B cell interactome has limitations. It was constructed using HG-U95Av2 Affymetrix microarrays probing relative expression of 12,625 genes. Additionally, the B cell interactome was constructed using an overwhelming proportion (92.6%) of a variety of neoplastic B cell types, including “34 samples of B cell chronic lymphocytic leukemia; 68 samples of diffuse large B cell lymphomas, including cases further classified as immunoblastic or centroblastic; 27 samples of Burkitt lymphoma; 6 samples of follicular lymphoma; 9 samples of primary effusion lymphoma; 8 samples of mantle cell lymphoma; 16 samples of hairy cell leukemia; 4 cell lines derived from Hodgkin disease; 5 B cell lymphoma cell lines; and 5 lymphoblastic cell lines. The data set also included a Burkitt lymphoma cell line (Ramos) treated in vitro to activate CD40 or BCR signaling and cell lines engineered to stably express BCL6 and BCL6(ΔPEST) mutant or to conditionally express BCL6 or MYC.”^90,91^

### Taiji

The Taiji software is an integrative multi-omics data analysis framework that integrates diverse data sets to construct regulatory networks and identify candidate driver genes. This package contains dependencies on the MACS2, BWA, and bedGraphToBigWig packages. Briefly, Taiji identifies active regulatory elements, including active promoters and active enhancers, defined by ATAC-seq peaks. Enhancers are then assigned to their promoters using EpiTensor predicted chromatin interactions. Transcriptional regulatory networks are constructed, by scanning regulatory elements for putative TF binding motifs supplied by the CIS-BP database, ultimately linking TFs to their target genes. Finally, the PageRank algorithm is used to assess the genome-wide influences of TFs. Furthermore, node and edge weights are used to personalize the ranking algorithm. Node weights are determined by the *z* scores of gene expression, ranking TFs higher if they regulate more differentially expressed genes. Edge weights are set to be proportional to a TFs’ expression levels, which filters out minimally expressed TFs. Thus, Taiji and the personalized PageRank analysis was performed using raw ATAC-seq data and RNA-seq gene expression tables comparing MBCs to NBCs. Only transcription factors with a PageRank score >0.05 in both sample groups were included to filter out less-important factors^92,93^.

### Statistical analysis and reproducibility

All statistical analyses were performed using Excel (Microsoft), GraphPad Prism, or R software environment. Differences in RNA transcript expression were determined by EdgeR, which implements statistical methods on empirical Bayes generalized linear models (GLMs) and determines differential expression through a pairwise *F*-test. Spearman correlation analysis was used to measure the strength and direction of mRNA-to-mRNA, mRNA-to-miRNA, mRNA-to-lncRNA, and miRNA-to-lncRNA expression correlations. Data are derived from three or more independent experiments.

### Reporting summary

Further information on research design is available in the Nature Research Reporting Summary linked to this article.

## Supplementary information

Supplementary Information

Reporting Summary

## Data Availability

All sequencing data that support the findings of this study have been made publicly available. Raw and processed data files for the RNA-Seq, smRNA-Seq, and ATAC-Seq have been deposited in the NCBI Gene Expression Omnibus under accession number GSE156904. Raw data files for recombined V_H_DJ_H_-C_H_ amplicons the have been deposited in the NCBI BioProject database under accession number PRJNA658698. No custom or modified scripts were used in association with this study. All other relevant data and materials are available from the corresponding author on request. Source data are provided with this paper.

## Source data

Source Data
