# Supplementary material for: Integrative transcriptome and chromatin landscape analysis reveals distinct epigenetic regulations in human memory B cells
Source: Nat Commun. 2020 Oct 28;11:5435. doi: 10.1038/s41467-020-19242-6 (PMC7595102; doi:10.1038/s41467-020-19242-6)
Supplement: Supplementary file 1 — Supplementary Information [file 41467_2020_19242_MOESM1_ESM.pdf]

SUPPLEMENTAL MATERIAL

Integrative transcriptome and chromatin landscape analysis reveals distinct epigenetic regulations in human memory B cells

Justin B. Moroney, Anusha Vasudev, Alexander Pertsemlidis, Hong Zan and Paolo Casali

Supplementary Figures

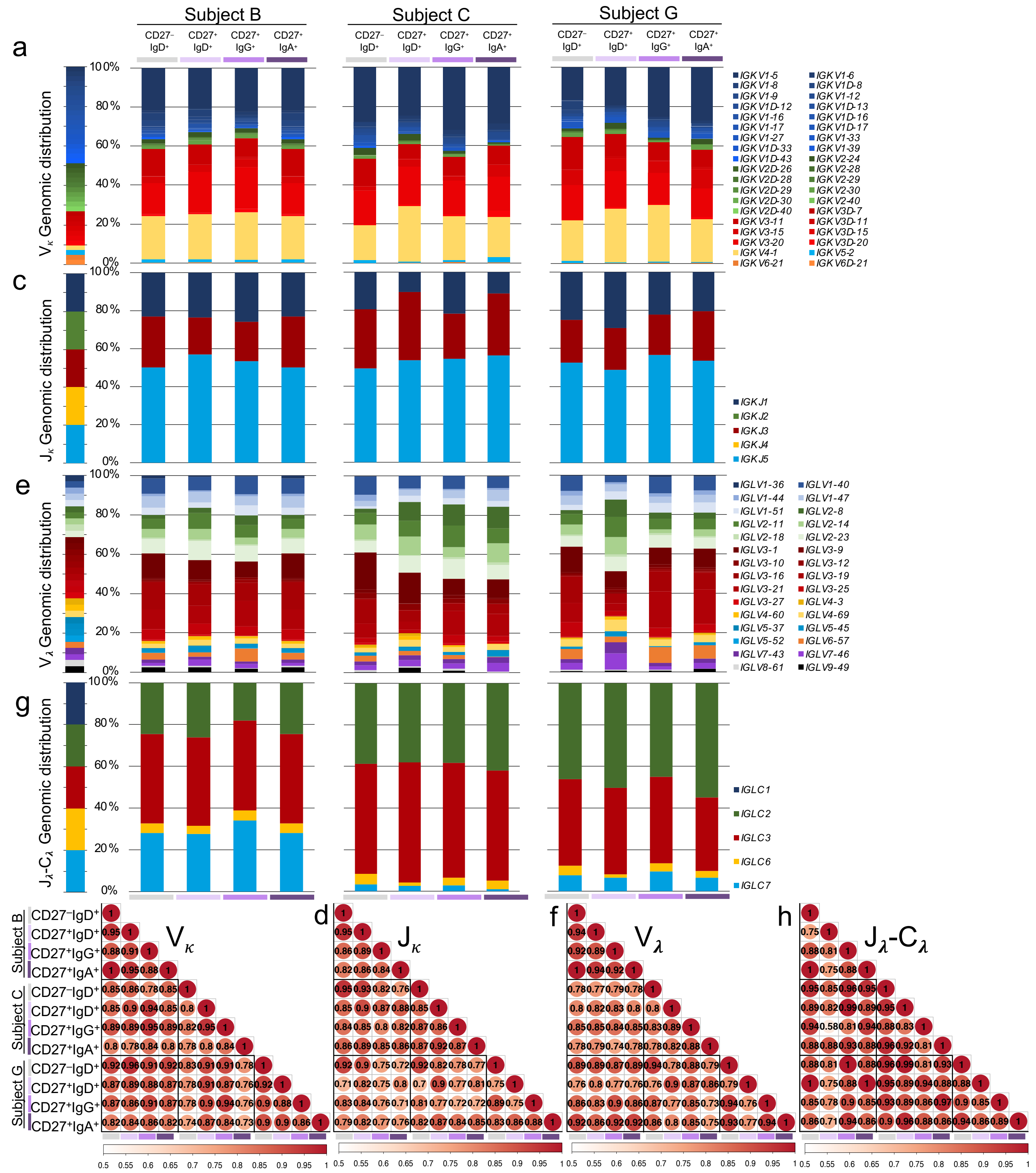

Supplementary Fig.1 Expressed Vκ, Jκ , Vλ and Jλ genes in human CD27⁻IgD⁺, CD27⁺IgD⁺, CD27⁺IgG⁺ and CD27⁺IgA⁺ B cells. (**a,c,e,g**), Ig Vκ(**a**), Jκ(**c**), Vλ(**e**) and Jλ-Cλ (**g**) gene expression in CD27⁻IgD⁺, CD27⁺IgG⁺, CD27⁺IgA⁺ and CD27⁺IgD⁺ B cells, as depicted by stacked column for each subject (subjects B, C, G). Gene members grouped within a family are depicted in shades of the respective family/group color. Vκ family: Vκ1 (blue), Vκ2 (green), Vκ3 (red), Vκ4 (yellow), Vκ5 (teal), Vκ6 (orange). Jκ genes: Jκ1 (orange), Jκ2 (teal), Jκ3 (yellow), Jκ4 (red), Jκ5 (blue). Vλ subgroups: Vλ1 (blue), Vλ2 (green), Vλ3 (red), Vλ4 (yellow), Vλ5 (teal), Vλ6 (orange), Vλ7 (purple), Vλ8 (grey), Vλ9 (black). Jλ-Cλ genes: Jλ1-Cλ1 (dark blue), Jλ2-Cλ2 (green), Jλ3-Cλ3 (red), Jλ6-Cλ6 (yellow), Jλ7-Cλ7 (teal). Jλ reads fall below the minimum expression threshold (>1 mapped read), thus Cλ expression was used as a surrogate for linked Jλ-Cλ usage.(**b,d,f,h**) Correlation matrix showing the relationship of individual Ig Vκ (**b**), Jκ (**d**), Vλ (**f**) and Jλ-Cλ (**h**) gene expression between B cell subsets in all 3 subjects. Color bars denote correlation coefficients on a scale from 0.5 (white) to 1 (red), with color, circle size and value all indicating the overall strength of the correlation. B cell subsets are repeated along bottom, left to right (grey, lavender, purple, dark purple).

a

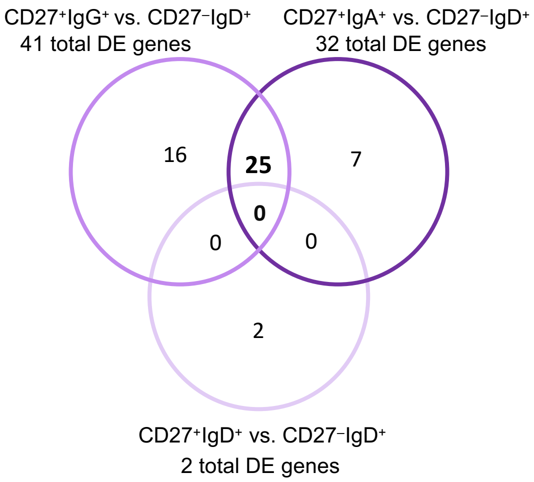

b

	IgG-specific genes	CD27+IgG+ (p_{adj})	CD27+IgA+ (p_{adj})		IgA-specific genes	CD27+IgG+ (p_{adj})	CD27+IgA+ (p_{adj})
1	*IGHG1*	6.81E-78	4.85E-13	1	*IGHA1*	2.86E-22	1.19E-35
2	*COCH*	1.84E-34	6.10E-16	2	*IGHA2*	1.63E-15	3.00E-84
3	*BTNL9*	5.87E-35	1.07E-19	3	*CD200*	3.34E-27	5.41E-38
4	*ATXN1*	1.47E-32	1.35E-19	4	*DSP*	2.80E-25	8.05E-48
5	*KLF4*	2.92E-43	1.52E-30	5	*COL18A1*	1.45E-23	6.18E-35
6	*NEK6*	2.97E-42	7.47E-26	6	*BCL7A*	3.36E-24	2.97E-33
7	*FAM129A*	2.02E-39	5.62E-25	7	*MXI1*	1.49E-22	2.10E-32
8	*XYLT1*	6.42E-39	2.43E-21				
9	*YBX3*	5.57E-37	2.03E-28				
10	*MCOLN2*	1.55E-36	1.46E-28				
11	*ACSM3*	3.10E-36	2.06E-21				
12	*ARHGAP31*	8.98E-32	2.22E-23				
13	*COL19A1*	8.98E-32	3.17E-30				
14	*HIPK2*	1.26E-31	1.83E-24				
15	*MMP17*	4.70E-31	1.87E-21				
16	*IL6*	7.39E-31	5.18E-19				

c

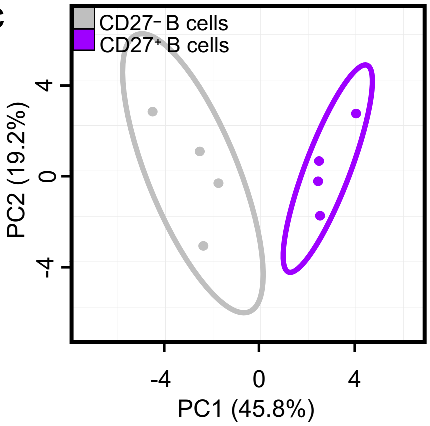

d

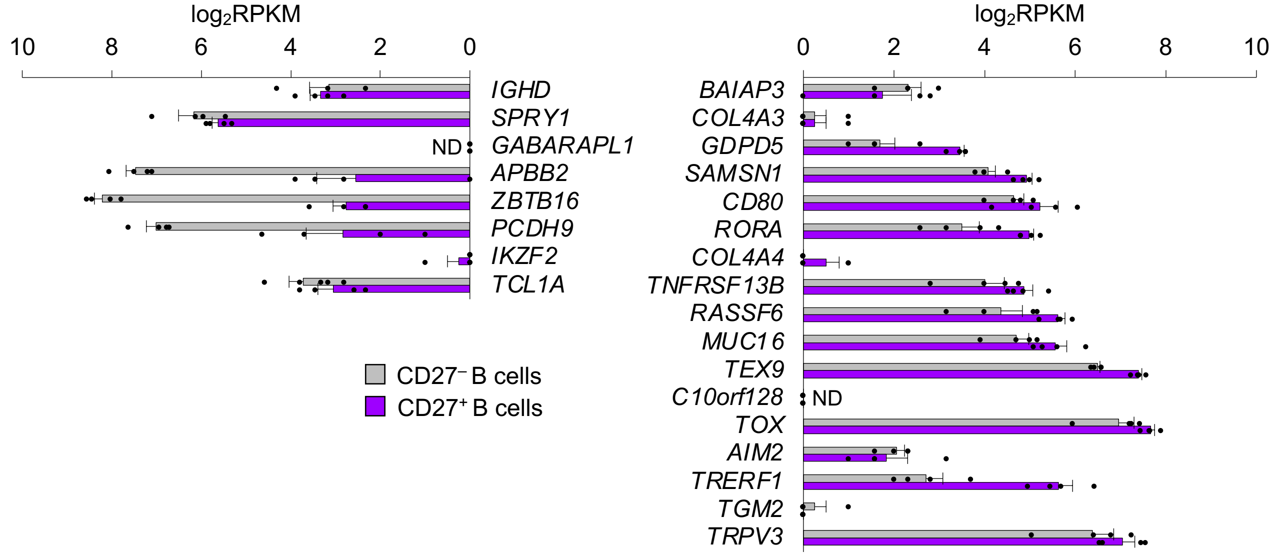

Supplementary Fig. 2 Transcriptome analysis of human B cell sub-subsets. a, Overlap of DE mRNA transcripts in $CD27^+$ B cell subsets, as compared to $CD27^-IgD^+$ B cells at a p_{adj} <10x10^{-30} depicted by Venn diagram. **b**, $CD27^+IgG^+$ and $CD27^+IgA^+$ swMBC isotype-specific DE gene expression, as compared to $CD27^-IgD^+$ NBCs at a p_{adj} <10x10^{-30} as determined by edgeR. **c**, Transcriptome clustering of $CD27^-$ and $CD27^+$ B cells was performed using the top 24 DE mRNA transcripts and depicted by PCA plot. Prediction ellipses denote 95% confidence intervals. Each symbol represents an individually sorted subset (n = 4). **d,** Normalized (log_2RPKM) expression of the 24 mRNAs that comprise the swMBC signature are depicted by histogram for $CD27^-$ and $CD27^+$ B cells. Data are mean ± SEM derived from analysis of B cells from subjects A, D, E, F (n = 4). ND, not determined.

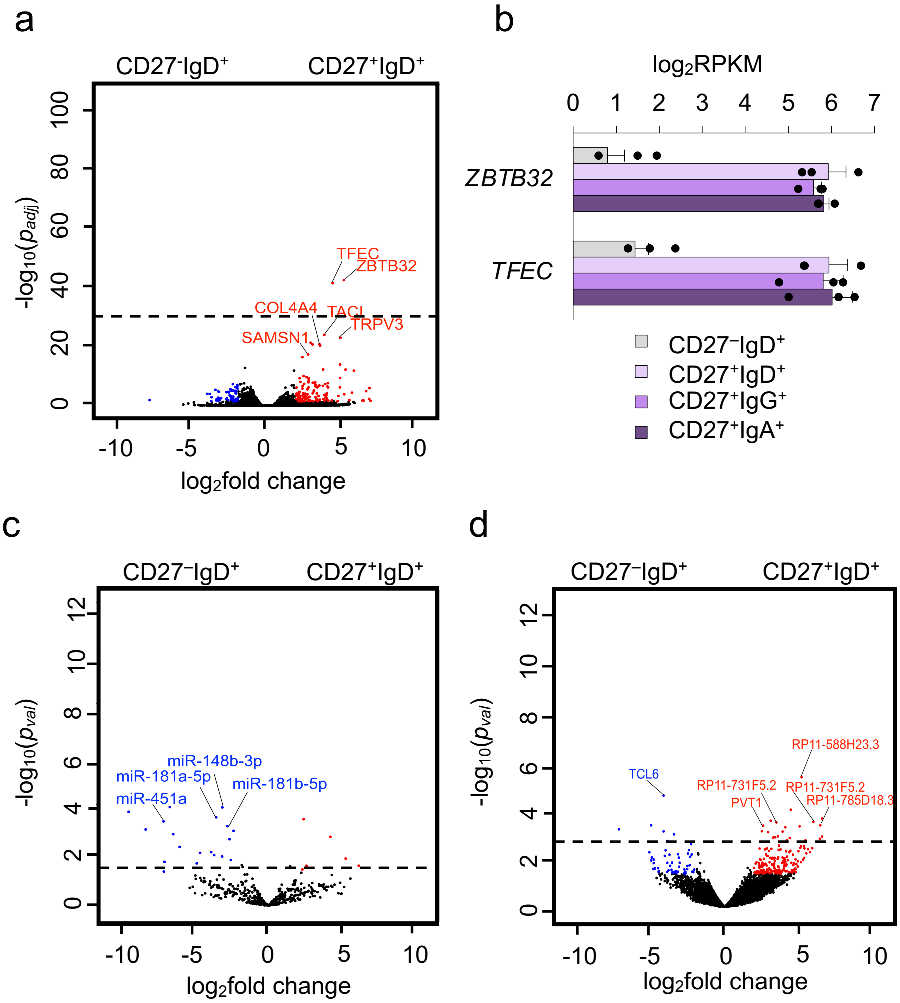

Supplementary Fig. 3 Transcriptome analysis of human CD27$^+$IgD$^+$ B cells. (**a**,**c**,**d**) Global transcriptional differences in mRNA (**a**), miRNA (**c**) and lncRNA (**d**) in CD27$^+$IgD$^+$ vs. CD27$^-$IgD$^+$ B cells (from subjects B, C, G; n = 3) as depicted by volcano plot. All genes annotated in human GENCODE v24 GRCh38 are shown, with each circle representing 1 gene. Significantly altered RNA transcripts are highlighted in red (upregulated) or in blue (downregulated). -Log$_{10}$-transformed p_{adj} is shown on the y-axis for mRNA and -log$_{10}$-transformed *p is* shown on the y-axis for miRNAs and lncRNAs. Log$_2$-transformed fold change of transcript expression is shown on the x-axis. DE mRNA, miRNA and lncRNA transcripts shared by swMBC signature and CD27$^+$IgD$^+$ B cells are annotated. **b**, Normalized (log$_2$RPKM) expression of the 2 most altered mRNA transcripts in CD27$^+$IgD$^+$ unswMBCs as compared to CD27$^-$IgD$^+$ NBCs, as depicted by histogram for each subset. Data are mean ± SEM derived from analysis of B cells from subjects B, C, G (n = 3).

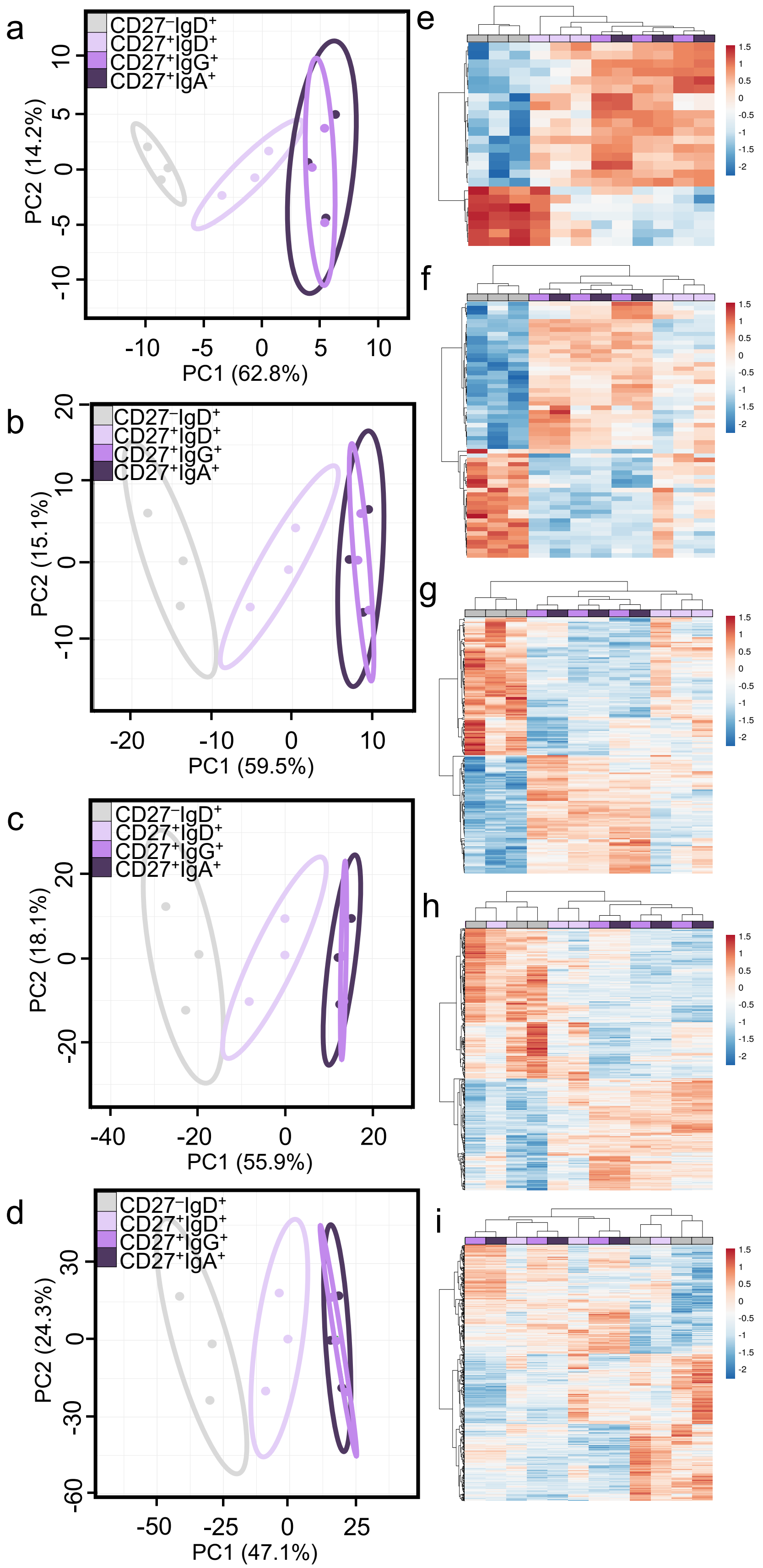

Supplementary Fig. 4 Principal Component Analysis and unsupervised Euclidean hierarchical clustering of expanded DE gene profiles a, Transcriptome clustering of the sorted B cell subsets performed using the 60 mRNAs DE between CD27$^+$IgG$^+$ and CD27$^+$IgA$^+$swMBCs vs. CD27$^-$IgD$^+$NBCs (p_{adj}<10x10^{-20}) is depicted by PCA plot (n = 3). **b,** Transcriptome clustering of the sorted B cell subsets performed using the 181 mRNAs DE between CD27$^+$IgG$^+$ and CD27$^+$IgA$^+$swMBCs vs. CD27$^-$IgD$^+$NBCs (p_{adj}<10x10^{-10}) is depicted by PCA plot (n = 3). **c,** Transcriptome clustering of the sorted B cell subsets performed using the 449 mRNAs DE between CD27$^+$IgG$^+$ and CD27$^+$IgA$^+$swMBCs vs. CD27$^-$IgD$^+$NBCs (p_{adj}<10x10^{-5}) is depicted by PCA plot (n = 3). **d,** Transcriptome clustering of the sorted B cell subsets performed using the 1074 mRNAs DE between CD27$^+$IgG$^+$ and CD27$^+$IgA$^+$swMBCs vs. CD27$^-$IgD$^+$NBCs (p_{adj}<0.05) is depicted by PCA plot (n = 3). For **a, b, c, d,** prediction ellipses define 95% confidence intervals. Each symbol represents an individually sorted subset (n = 3). DEGs as determined by edgeR. **e,** Unsupervised hierarchical clustering of the sorted B cell subsets performed using the 24 mRNAs DE between CD27$^+$IgG$^+$ and CD27$^+$IgA$^+$swMBCs vs. CD27$^-$IgD$^+$NBCs (p_{adj}<10x10^{-30}) is depicted by heatmap and dendrogram. **f,** Unsupervised hierarchical clustering of the sorted B cell subsets performed using the 60 mRNAs DE between CD27$^+$IgG$^+$ and CD27$^+$IgA$^+$swMBCs vs. CD27$^-$IgD$^+$NBCs (p_{adj}<10x10^{-20}) is depicted by heatmap and dendrogram. **g,** Unsupervised hierarchical clustering of the sorted B cell subsets performed using the 181 mRNAs DE between CD27$^+$IgG$^+$ and CD27$^+$IgA$^+$swMBCs vs. CD27$^-$IgD$^+$NBCs (p_{adj}<10x10^{-10}) is depicted by heatmap and dendrogram. **h,** Unsupervised hierarchical clustering of the sorted B cell subsets performed using the 449 mRNAs DE between CD27$^+$IgG$^+$ and CD27$^+$IgA$^+$swMBCs vs. CD27$^-$IgD$^+$NBCs (p_{adj}<10x10^{-5}) is depicted by heatmap and dendrogram. **i,** Unsupervised hierarchical clustering of the sorted B cell subsets performed using the 1074 mRNAs DE between CD27$^+$IgG$^+$ and CD27$^+$IgA$^+$swMBCs vs. CD27$^-$IgD$^+$NBCs (p_{adj}<0.05) is depicted by heatmap and dendrogram. For **e, f, g, h, i,** unsupervised hierarchical clustering was performed using Euclidean distance and average linkage, with vertical distances on each branch of the dendrogram representing the degree of similarity between gene expression profiles of the 4 MBC groups. DEGs as determined by edgeR.

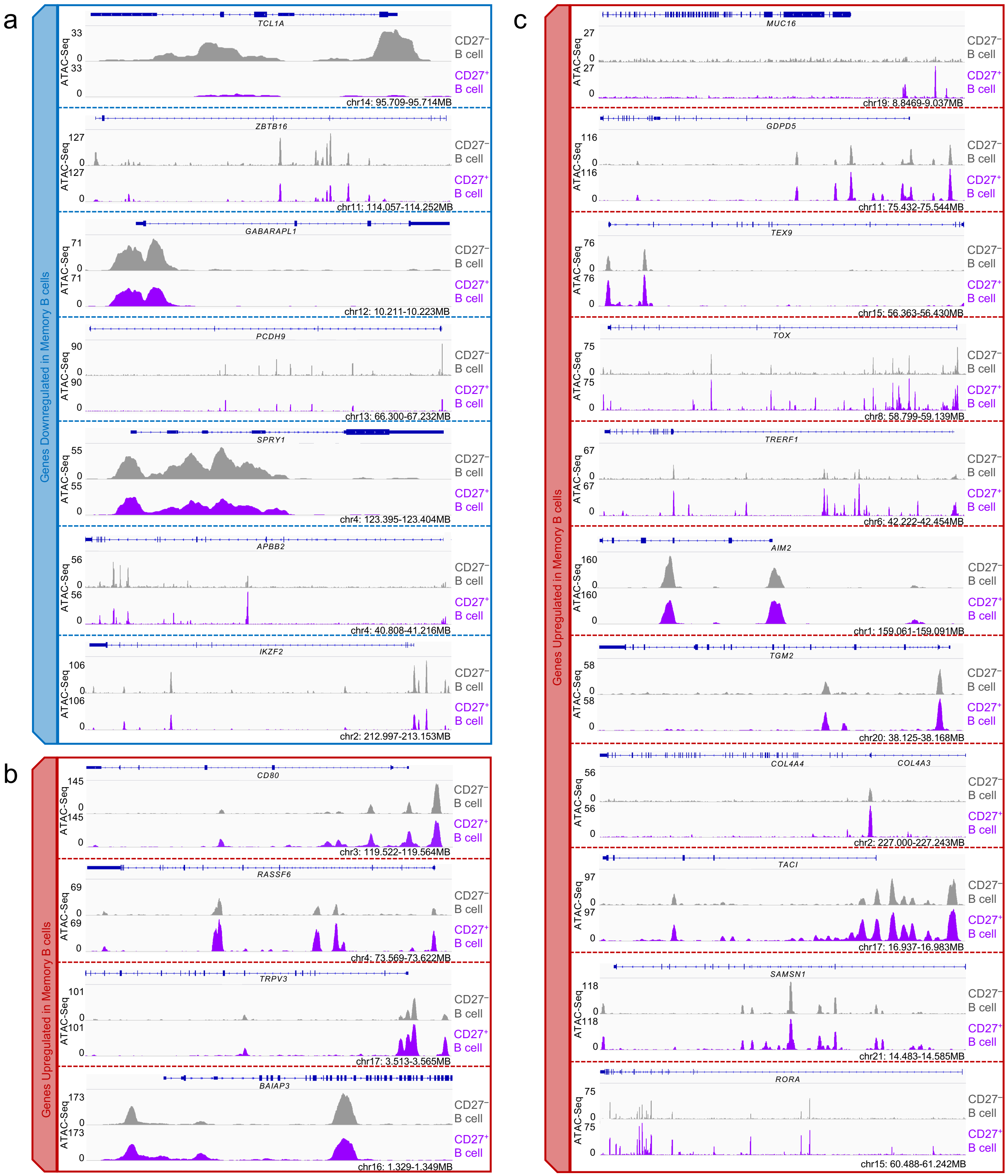

Supplementary Fig. 5 Chromatin accessibility of signature genes in human swMBCs. a-c Chromatin accessibility of human swMBC signature genes with decreased (**a**) and increased (**b**,**c**) expression is displayed by IGV gene tracks. Coverage includes gene introns, exons, promoter regions and potential enhancer regions. ATAC-Seq signal is normalized for the window of interest. NBCs depicted in grey and all MBCs in purple.

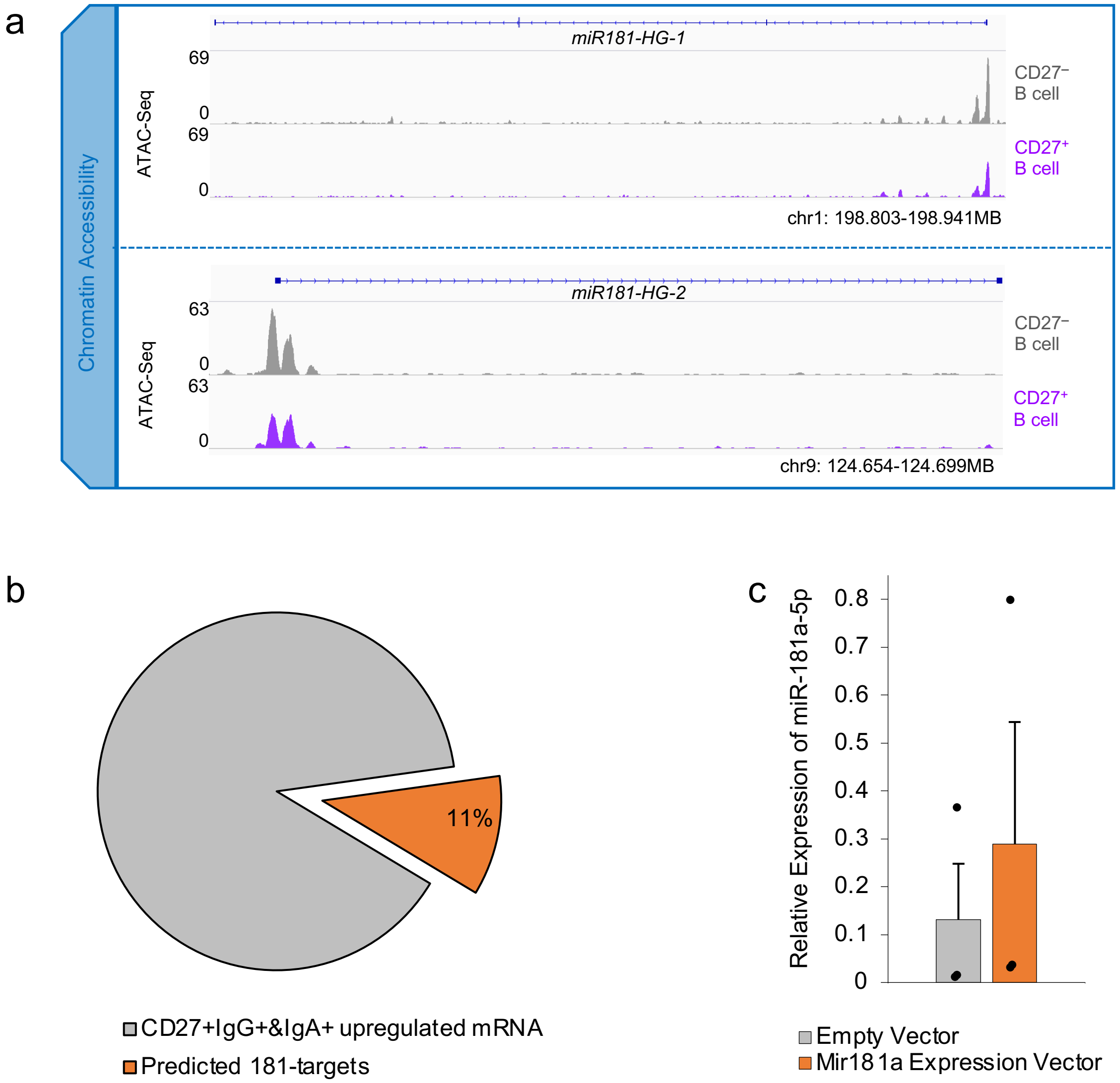

Supplementary Fig. 6 MIR181 as a regulator of swMBC-specific gene expression. **a,** Chromatin accessibility of MIR181 host genes on chromosomes 1 and 9 as depicted by IGV gene track. Coverage includes gene introns, exons, promoter regions and potential enhancer regions. ATAC-Seq signal is normalized for the window of interest, with NBCs depicted in grey and all MBCs in purple. **b,** The percentage of mRNAs upregulated in swMBCs ($CD27^+IgG^+$ and $CD27^+IgA^+$; $p_{adj}<0.05$) that are predicted targets of MIR181a and MIR181b is depicted by the pie chart (50/462 mRNAs). **c,** Mir181a-5p expression (qPCR) in sorted ($7AAD^-$ $CD19^+GFP^+$) B cells transduced by MDH1-PGK-GFP (Empty Vector, n = 3) or MDH1-miR-181a-1-PGK-GFP (Mir181a Expression Vector, n = 3) retrovirus. Data are mean ± SEM.

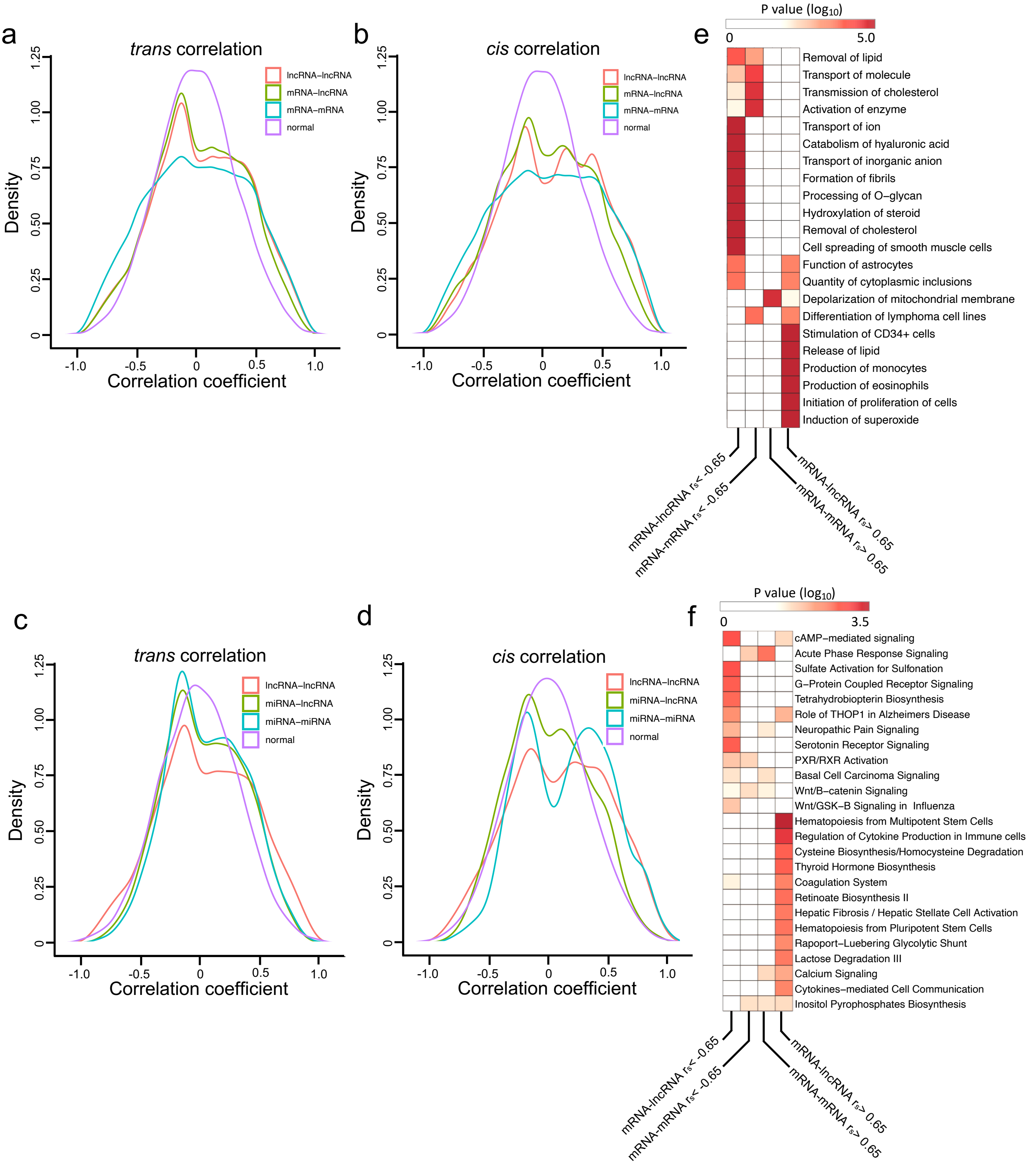

Supplementary Fig. 7 Correlation of RNA species across human NBCs and MBCs. (**a**,**b**) Spearman correlations between RNA species (*trans*, genome-wide pairs; *cis*, pairs within 1Mb) plotted by density histogram. lncRNA-lncRNA correlations, red; mRNA-lncRNA correlations, green; mRNA-mRNA correlations, blue; normal distribution, purple. (**c**,**d**) Spearman correlations between RNA species (trans, genome-wide pairs; cis, pairs within 1Mb) plotted by density histogram. lncRNA-lncRNA correlations, red; miRNA-lncRNA correlations, green; miRNA-miRNA correlations, blue; normal distribution, purple. (**e**,**f**) Gene ontology and pathway enrichment analysis of mRNA with strong ($r_s \pm 0.65$) *cis* Spearman correlations to lncRNA or mRNA transcripts determined by over-representation analysis in IPA. -Log_{10}-transformed *p* of the most significant categories displayed by heatmap.

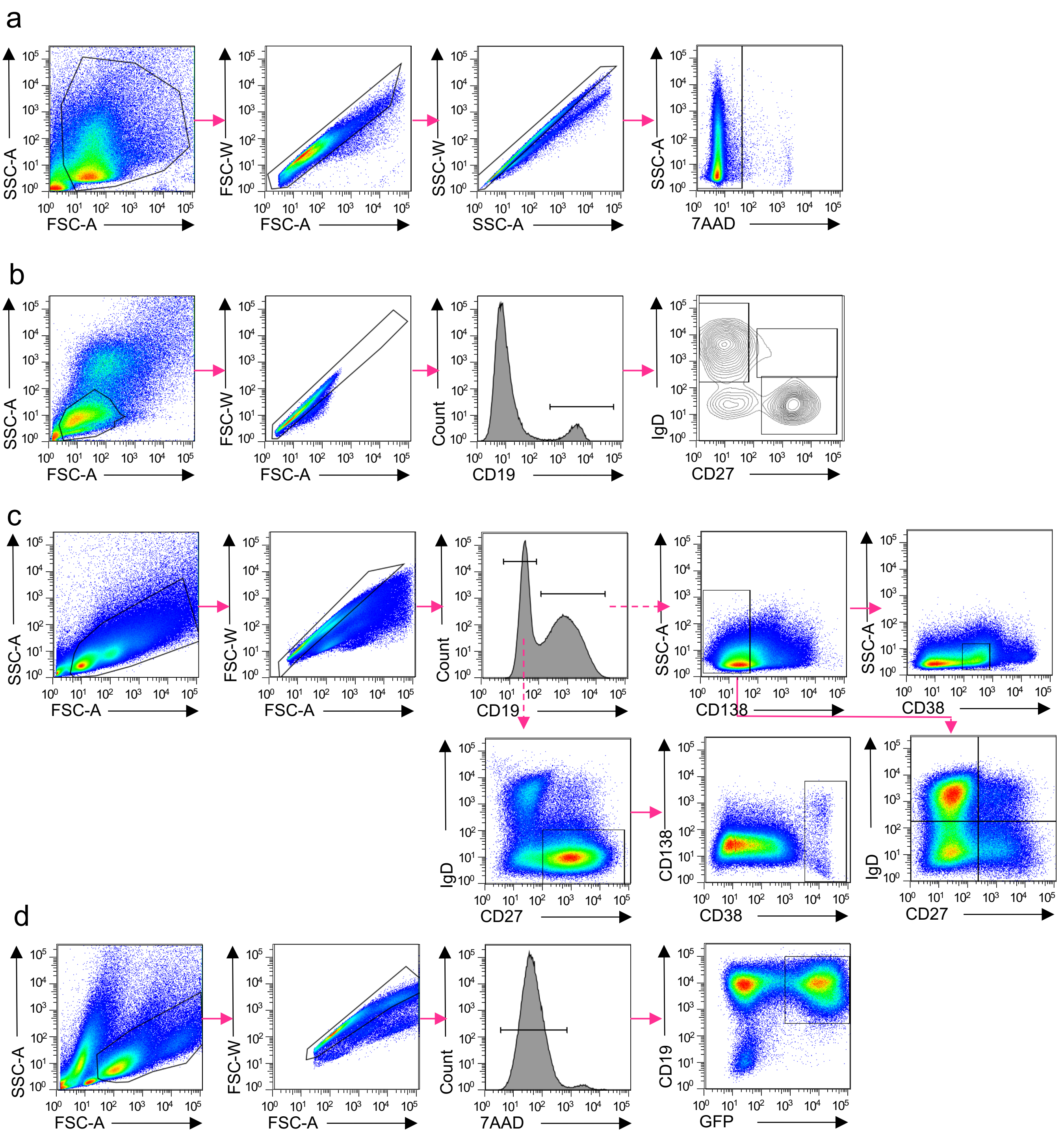

Supplementary Figure 8. Gating strategy for flow cytometry analysis. a, Gating strategy to analyze and sort NBCs, class switched MBCs and unswitched MBCs Fig. 1. **b**. Gating strategy to analyze intracellular RORα expression in peripheral blood B cells presented in Fig. 5e. **c**, Gating strategy to analyze intracellular RORα expression in tonsil B cells presented in Fig. 5f. **d**, Gating strategy to analyze and sort retrovirally transduced B cells Fig. 8c.

Supplementary Tables

Supplementary Table 1. Human subjects (7) studied.

Subject	Age	Sex	Race Ethnicity	CD27⁻IgD⁺	CD27⁺IgD⁺	CD27⁺IgG⁺	CD27⁺IgA⁺	B cell isolation	ATAC	mRNA	miRNA	lncRNA
A†	20	Female	White	73.2%	3.50%	5.81%	4.20%	CD27-<br>total CD27+	Yes	Yes	---	---
B*	20	Female	Multi-racial	52.6%	15.9%	12.57%	7.37%	CD27- IgD+<br>CD27+IgD+<br>CD27+IgG+<br>CD27+IgA+	---	Yes	Yes	Yes
C*	21	Male	White	78.8%	6.28%	3.62%	2.54%	CD27- IgD+<br>CD27+IgD+<br>CD27+IgG+<br>CD27+IgA+	---	Yes	Yes	Yes
D†	33	Male	White	45.3%	25.6%	8.14%	5.58%	CD27-<br>total CD27+	Yes	Yes	---	---
E†	35	Female	White Hispanic	50.1%	2.36%	5.86%	7.79%	CD27-<br>total CD27+	Yes	Yes	---	---
F†	36	Male	African American	68.2%	5.75%	5.88%	4.13%	CD27-<br>total CD27+	Yes	Yes	---	---
G*	39	Male	White	79.2%	8.19%	3.66%	2.99%	CD27- IgD+<br>CD27+IgD+<br>CD27+IgG+<br>CD27+IgA+	---	Yes	Yes	Yes
**Mean**	29.1 ±8.4	3F/4M	N/A	63.9±14.3%	9.7±8.3%	6.5±3.1%	4.9±2.0%	N/A	N/A	N/A	N/A	N/A

* Subjects B, C, G: 4 B cell fractions sorted (CD27⁻IgD⁺, CD27⁺IgD⁺, CD27⁺IgG⁺, and CD27⁺IgA⁺), mRNA, miRNA, lncRNA sequencing.

† Subjects A, D, E, F: 2 B cell fractions sorted (CD27⁻ and CD27⁺), mRNA sequencing, ATAC (assay for transposase accessible chromatin).

Supplementary Table 2. Memory B cell gene functions.

Upregulated Genes			
**Gene Name**	**Protein Identifier**	**Localization**	**Function**
*MUC16**	Mucin 16	PM	Adhesion molecule/signal transduction
*GDPD5**	Glycerophosphodiester Phosphodiesterase Domain Containing 5	PM/ER	Lipid metabolism
*TEX9**	Testis Expressed 9	C	Unknown
*TOX**	Thymocyte Selection Associated High Mobility Group Box	N	High motiliy group-box transcription factor
*TRERF1**	Transcriptional Regulating Factor 1	N/C	Zinc-finger transcription factor
*AIM2**	Absent In Melanoma 2	M/N/C	Inflammasome activation
*TGM2**	Transglutaminase 2	PM/EC/M/C	Calcium-dependent protein crosslinking
*C10orf128**	Chromosome 10 Open Reading Frame 128	PM	Unknown function
*RASSF6**	Ras Association Domain Family Member 6	N/C	Tumor suppressor/Self-renwal signaling
*TRPV3**	Transient Receptor Potential Cation Channel Subfamily V Member 3	PM	Calcium-permeable temperature-sensitive cation channel
BAIAP3	BAI1 Associated Protein 3	ER	Calcium stimulated exocytosis
COL4A3	Collagen Type IV Alpha 3 Chain	EC/ER/N	Structural component
TNFRSF13B	TNF Receptor Superfamily Member 13B (TACI)	PM	BAFF/APRIL receptor
SAMSN1	SAM Domain, SH3 Domain And Nuclear Localization Signals 1	N/C	Negative regulator of B-cell activation.
CD80	B7-1 Antigen	PM	Co-stimulatory Molecule
RORA	Retinoic Acid Receptor-Related Orphan Receptor Alpha	N/C	Transcription factor
COL4A4	Collagen Type IV Alpha 4 Chain	EC/ER/N	Structural component
**Downregulated Genes**			
**Gene Name**	**Protein Identifier**	**Localization**	**Function**
*IGHD**	Immunoglobulin Heavy Constant Delta	PM/EC	Immunoglobulin constant region
*APBB2**	Amyloid Beta Precursor Protein Binding Family B Member 2	N/M/C	Signal transduction molecule
*TCL1A**	T Cell Leukemia/Lymphoma 1A	N/ER	Signal transduction
IKZF2	IKAROS Family Zinc Finger 2(Helios)	N	Transcription factor
ZBTB16	Zinc Finger And BTB Domain Containing 16 (PLZF)	N/C	Kruppel-like Transcription factor
PCDH9	Protocadherin 9	PM	Calcium-dependent adhesion molecule
SPRY1	Sprouty RTK Signaling Antagonist 1	PM/N/C/GA	Antagonist of B cell proliferation signaling
GABARAPL1	GABA Type A Receptor Associated Protein Like 1	C/ER/GA	Autophagy

PM=Plasma Membrane; ER=Endoplasmic Reticulum; N=Nucleus; C=cytosol; EC=Extracellular; GA=Golgi Apparatus; M=mitochondria .
Localization ordered according to UniProt confidence
Genes annotated with “ * ” are differentially expressed with a Fold change >±32

Supplementary Table 3. Pairwise gene expression correlation analysis

Correlation Type	Pair class	Total number of correlations	Spearman correlation threshold: percent of pairs									
			rho>0	rho>0.1	rho>0.2	rho>0.3	rho>0.4	rho>0.5	rho>0.6	rho>0.7	rho>0.8	rho>0.9
			rho<0	rho<-0.1	rho<-0.2	rho<-0.3	rho<-0.4	rho<-0.5	rho<-0.6	rho<-0.7	rho<-0.8	rho<-0.9
Trans												
	mRNA-mRNA	205093101										
			50.978	43.830	36.572	29.361	21.507	14.788	9.580	5.203	2.167	0.439
			49.022	41.237	33.585	26.376	18.840	12.990	8.412	4.571	1.865	0.331
	lncRNA-lncRNA	2080054										
			53.211	45.873	38.212	30.375	21.734	14.308	9.285	5.041	2.056	0.408
			46.789	37.545	27.964	20.090	12.456	8.005	5.048	2.662	0.986	0.143
	miRNA-miRNA	26191										
			53.297	44.561	35.520	25.826	16.582	9.047	5.040	2.130	0.540	0.084
			46.703	35.959	23.218	13.974	6.819	3.394	1.546	0.530	0.180	0.011
	mRNA-lncRNA	8717824										
			51.248	43.485	35.405	27.347	18.708	11.392	6.756	3.294	1.123	0.168
			48.753	39.081	29.041	20.663	12.573	7.952	4.930	2.600	0.968	0.146
	mRNA-miRNA	4621095										
			49.909	40.902	31.873	23.193	14.479	8.037	4.048	1.580	0.400	0.042
			50.091	40.055	29.607	20.507	11.754	6.194	3.024	1.160	0.290	0.030
	miRNA-lncRNA	455194										
			51.942	43.299	34.325	25.186	16.025	9.066	4.707	1.840	0.42	0.046
			48.058	37.758	26.113	16.879	8.763	4.425	2.074	0.780	0.19	0.013
Cis												
	mRNA-mRNA	291958										
			54.017	47.277	40.298	33.274	25.635	18.706	12.955	7.651	3.6402	0.9539
			45.983	38.657	31.603	24.913	18.091	12.543	8.094	4.375	1.7931	0.30792
	lncRNA-lncRNA	2842										
			57.002	50.281	42.927	34.588	26.950	18.508	12.491	7.424	3.131	0.809
			42.998	35.362	26.249	19.493	12.421	7.987	4.821	2.322	0.739	0.105
	miRNA-miRNA	234										
			59.402	55.983	47.436	38.034	28.632	20.512	12.393	7.690	4.270	0.855
			40.598	32.051	20.940	13.248	3.846	2.136	1.282	0.430	0	0
	mRNA-lncRNA	9592										
			52.637	44.995	36.853	28.774	20.558	12.875	8.110	3.972	1.574	0.260
			47.362	37.979	29.232	21.820	13.667	8.892	5.535	3.054	1.105	0.135
	mRNA-miRNA	7148										
			49.077	39.647	30.162	22.440	14.088	7.946	4.155	1.510	0.340	0.028
			50.923	40.697	30.918	21.600	12.927	7.302	3.945	1.550	0.410	0.042
	miRNA-lncRNA	596										
			49.329	40.604	31.711	23.154	15.101	8.389	3.691	1.680	0.500	0
			50.671	40.940	28.188	20.302	11.577	6.208	2.181	0.670	0.170	0
