# Supplementary material for: Integrative transcriptome and chromatin landscape analysis reveals distinct epigenetic regulations in human memory B cells
Source: Nat Commun. 2020 Oct 28;11:5435. doi: 10.1038/s41467-020-19242-6 (PMC7595102; doi:10.1038/s41467-020-19242-6)
Supplement: Supplementary file 2 — Reporting Summary [file 41467_2020_19242_MOESM2_ESM.pdf]

nature research

Corresponding author(s): Paolo Casali, Hong Zan

Last updated by author(s): Sep 21, 2020

Reporting Summary

Nature Research wishes to improve the reproducibility of the work that we publish. This form provides structure for consistency and transparency in reporting. For further information on Nature Research policies, see Authors & Referees and the Editorial Policy Checklist .

Statistics

For all statistical analyses, confirm that the following items are present in the figure legend, table legend, main text, or Methods section.

n/a	Confirmed	
☐	☒	The exact sample size (n) for each experimental group/condition, given as a discrete number and unit of measurement
☐	☒	A statement on whether measurements were taken from distinct samples or whether the same sample was measured repeatedly
☐	☒	The statistical test(s) used AND whether they are one- or two-sided<br>*Only common tests should be described solely by name; describe more complex techniques in the Methods section.*
☒	☐	A description of all covariates tested
☒	☐	A description of any assumptions or corrections, such as tests of normality and adjustment for multiple comparisons
☐	☒	A full description of the statistical parameters including central tendency (e.g. means) or other basic estimates (e.g. regression coefficient) AND variation (e.g. standard deviation) or associated estimates of uncertainty (e.g. confidence intervals)
☐	☒	For null hypothesis testing, the test statistic (e.g. F, t, r) with confidence intervals, effect sizes, degrees of freedom and P value noted<br>*Give P values as exact values whenever suitable.*
☒	☐	For Bayesian analysis, information on the choice of priors and Markov chain Monte Carlo settings
☒	☐	For hierarchical and complex designs, identification of the appropriate level for tests and full reporting of outcomes
☒	☐	Estimates of effect sizes (e.g. Cohen's d, Pearson's r), indicating how they were calculated

Our web collection on statistics for biologists contains articles on many of the points above.

Software and code

Policy information about availability of computer code

Data collection

- Quant Studio Real-Time PCR Software v1.5.1
- BD FACSDiva Software version 8.0.1
- FlowJo Software version 10.6.2
- Illumina HiSeq 3000 version HCS 3.4.0
- Illumina MiSeq version MCS v3.1
- Microsoft Excel version 16.33 (20011301)
- GraphPad Prism version 8.3.0

Data analysis

- Trim Galore (version 0.6.4)
- Hisat2 (version 2.1.0)No custom or modified scripts were used in association with this study.
- Samtools (version 1.10)
- Bedtools (2.28.0)
- HTSeq (version 0.11.13)
- EdgeR(version 3.10)
- Clustvis(version 1)
- R(version 3.3.3)
- Ingenuity Pathway analysis (Qiagen; Winter 2019 release)
- Taiji (version 1.2.1)
- MACS2 (version 2.2.7.1)
- BWA (version 0.5.9)
- bedGraphToBigWig (version 302)
- DESeq2(version 3.10)
- DAStk(version 1.0.0)

- HOMER(version 4.11)
- MARINa (version 1.0.0)
- VIPER (version 1.22.0)
- B cell Interactome (version 1.0.0)
All analyses are documented on the manuscript. No custom or modified scripts were used in association with this study.

For manuscripts utilizing custom algorithms or software that are central to the research but not yet described in published literature, software must be made available to editors/reviewers. We strongly encourage code deposition in a community repository (e.g. GitHub). See the Nature Research guidelines for submitting code & software for further information.

Data

Policy information about availability of data

All manuscripts must include a data availability statement. This statement should provide the following information, where applicable:

- Accession codes, unique identifiers, or web links for publicly available datasets
- A list of figures that have associated raw data
- A description of any restrictions on data availability

All sequencing data that support the findings of this study have been made publicly available. Raw and processed data files for the RNA-Seq, smRNA-Seq and ATAC-Seq have been deposited in the NCBI Gene Expression Omnibus under accession number GSE156904 (https://www.ncbi.nlm.nih.gov/geo/query/acc.cgi?acc=GSE156904). Raw data files for recombined VHDJH-CH amplicons the have been deposited in the NCBI BioProject database under accession number PRJNA658698 (https://www.ncbi.nlm.nih.gov/bioproject/658698). No custom or modified scripts were used in association with this study. All other relevant data are available from the corresponding author on request.

Field-specific reporting

Please select the one below that is the best fit for your research. If you are not sure, read the appropriate sections before making your selection.

☒ Life sciences ☐ Behavioural & social sciences ☐ Ecological, evolutionary & environmental sciences

For a reference copy of the document with all sections, see nature.com/documents/nr-reporting-summary-flat.pdf

Life sciences study design

All studies must disclose on these points even when the disclosure is negative.

Sample size

No sample-size calculations were performed. Sample sizes were chosen to provide enough replicates for statistical analysis and were determined by availability of biological samples from the South Texas Blood and Tissue Center, San Antonio, TX and based on ours and others experience with biological samples. See relevant literature below:

- Sanchez HN, Moroney JB, Gan H, Shen T, Im JL, Li T, Taylor JR, Zan H, Casali P. B cell-intrinsic epigenetic modulation of antibody responses by dietary fiber-derived short-chain fatty acids. Nat Commun. 2020 Jan 2;11(1):60. doi: 10.1038/s41467-019-13603-6. PMID: 31896754; PMCID: PMC6940392.
- Pone EJ, Lam T, Lou Z, Wang R, Chen Y, Liu D, Edinger AL, Xu Z, Casali P. B cell Rab7 mediates induction of activation-induced cytidine deaminase expression and class-switching in T-dependent and T-independent antibody responses. J Immunol. 2015 Apr 1;194(7):3065-78. doi: 10.4049/jimmunol.1401896. Epub 2015 Mar 4. PMID: 25740947; PMCID: PMC4643723.
- Gan H, Shen T, Chupp DP, Taylor JR, Sanchez HN, Li X, Xu Z, Zan H, Casali P. B cell Sirt1 deacetylates histone and non-histone proteins for epigenetic modulation of AID expression and the antibody response. Sci Adv. 2020 Apr 1;6(14):eaay2793. doi: 10.1126/sciadv.aay2793. PMID: 32270032; PMCID: PMC7112761.
- Scharer CD, Blalock EL, Barwick BG, Haines RR, Wei C, Sanz I, Boss JM. ATAC-seq on biobanked specimens defines a unique chromatin accessibility structure in naïve SLE B cells. Sci Rep. 2016 Jun 1;6:27030. doi: 10.1038/srep27030. PMID: 27249108; PMCID: PMC4888756.
- Barwick BG, Scharer CD, Bally APR, Boss JM. Plasma cell differentiation is coupled to division-dependent DNA hypomethylation and gene regulation. Nat Immunol. 2016 Oct;17(10):1216-1225. doi: 10.1038/ni.3519. Epub 2016 Aug 8. PMID: 27500631; PMCID: PMC5157049.
- Guo M, Price MJ, Patterson DG, Barwick BG, Haines RR, Kania AK, Bradley JE, Randall TD, Boss JM, Scharer CD. EZH2 Represses the B Cell Transcriptional Program and Regulates Antibody-Secreting Cell Metabolism and Antibody Production. J Immunol. 2018 Feb 1;200(3):1039-1052. doi: 10.4049/jimmunol.1701470. Epub 2017 Dec 29. PMID: 29288200; PMCID: PMC5780247.

Data exclusions

No data exclusion.

Replication

All figure legends of experimental data contain clear descriptions of the biological and technical replicates.

Randomization

The samples analyzed in this study were randomly chosen.

Blinding

Blinding was not performed as the results are directly derived from the sequencing analysis of sorted B cell populations, therefore each exact sample needed to be accounted for and paired with its intra-subject comparator.

Reporting for specific materials, systems and methods

We require information from authors about some types of materials, experimental systems and methods used in many studies. Here, indicate whether each material, system or method listed is relevant to your study. If you are not sure if a list item applies to your research, read the appropriate section before selecting a response.

Materials & experimental systems

n/a	Involved in the study
☐	☒ Antibodies
☐	☒ Eukaryotic cell lines
☒	☐ Palaeontology
☒	☐ Animals and other organisms
☐	☒ Human research participants
☒	☐ Clinical data

Methods

n/a	Involved in the study
☒	☐ ChIP-seq
☐	☒ Flow cytometry
☒	☐ MRI-based neuroimaging

Antibodies

Antibodies used	Antibodies for flow cytometry and FACS: PEcy7-anti-human-CD19 mAb (clone H1B19; Biolegend; cat# 302215), PE-anti-human-CD27 mAb (clone M-T271; Biolegend; cat# 356406), BV421-anti-human-IgD mAb (clone IA6-2; Biolegend; cat# 348226), FITC-anti-human-IgG mAb (clone G18-145; BD Pharmingen; cat# 560952), APC-anti-human-IgA mAb (clone IS11-8E10; Miltenyi Biotec; cat# 130-093-073), APC-Cy7-anti-IgM mAb (clone MHM-88, Biolegend; cat# 314520), APC-Cyanine7-anti-human-CD27 mAb (clone M-T271; Biolegend; cat# 356424), BV510-anti-human-CD138 mAb (clone MI15 Biolegend; cat# 356518), BV650-anti-human-CD38 mAb (clone HB-7; Biolegend; cat# 356620) and PE-anti-human-RORa mAb (Clone 784652; R&D Systems; cat# IC8924P-025).<br><br>Antibodies involved in negative selection of human B cells (included in EasySep™ Human Total B Cell Isolation cocktail, STEMCELL Technologies) were: anti-CD4, anti-CD8, anti-CD14, anti-CD16, anti-CD25, anti-CD36, anti-CD56, anti-CD61, anti-CD66b, anti-CD123 and anti-glycophorin A mAb-tetramer-conjugated to anti-dextran Abs, pull-down by dextran-coated magnetic particles (Proprietary kit, no other information known).
Validation	All antibodies were validated according to manufacturer information. These products lots have passed BioLegend/BD Pharmigen/Miltenyi Biotec/R&D Systems QC testing and is certified for use.

Eukaryotic cell lines

Policy information about cell lines

Cell line source(s)	HEK293T & CL-01 cells
Authentication	Authentication of HEK293T cell line was performed by the ATCC Cell Biology Collection, where this cell line was purchased. Authentication of CL-01 cell line was performed by our lab, where this cell line was created. This has recently been authenticated through flow cytometry of this cell lines class-switching as well as recombined IgH transcripts.<br>See publication for full details - (Cerutti A., Zan H., Schaffer A., Bergsagel L., Harindranath N., Max E.E., Casali P.<br>CD40 ligand and appropriate cytokines induce switching to IgG, IgA, and IgE and coordinated germinal center and plasmacytoid phenotypic differentiation in a human monoclonal IgM+IgD+ B cell line.<br>J. Immunol. 160:2145-2157(1998))
Mycoplasma contamination	These cell lines were not tested for mycoplasma contamination.
Commonly misidentified lines (See ICLAC register)	Not relevant to this study.

Human research participants

Policy information about studies involving human research participants

Population characteristics	Blood donors were seven human subjects (20 and 39 years of age; 4 males and 3 females) healthy and non-immuno-compromised.
Recruitment	Buffy coats were obtained from the South Texas Blood and Tissue Center, San Antonio, TX. No self-selection bias or other biases were known to be present as human subjects were chosen based on buffy coat availability, which is determined by South Texas Blood and Tissue Center.
Ethics oversight	Blood collection was covered by an IRB protocol of the South Texas Blood and Tissue Center, San Antonio, TX. All experiments involving human samples were approved by the Institutional Review Board (IRB) of UTHSCSA, protocol #HSC20140234H (please see attached IRB approval)

Note that full information on the approval of the study protocol must also be provided in the manuscript.

Flow Cytometry

Plots

Confirm that:

- [x] The axis labels state the marker and fluorochrome used (e.g. CD4-FITC).
- [x] The axis scales are clearly visible. Include numbers along axes only for bottom left plot of group (a 'group' is an analysis of identical markers).
- [x] All plots are contour plots with outliers or pseudocolor plots.
- [x] A numerical value for number of cells or percentage (with statistics) is provided.

Methodology

Sample preparation	For flow cytometry analyses, PBMCs and B cells (isolated with EasySep™ Human Total B Cell Isolation Kit (STEMCELL Technologies) were stained with PEcy7-anti-human-CD19 mAb (clone H1B19; Biolegend), PE-anti-human-CD27 mAb (clone M-T271; Biolegend), BV421-anti-human-IgD mAb (clone HB-7; Biolegend), FITC-anti-human-IgG mAb (clone G18-145; BD Pharmingen), APC-anti-human-IgA mAb (clone IS11-8E10; Miltenyi Biotec) and APC-Cy7-anti-IgM mAb (clone MHM-88, Biolegend). Approximately 5 x 10^7 total B cells enriched from PBMCs were used for cell sorting. 75% of the total B cells were stained with FITC-anti-human-IgG mAb (clone G18-145; BD Pharmingen), APC-anti-human-IgA mAb (clone IS11-8E10; Miltenyi Biotec) and PE-anti-human-CD27 mAb (clone M-T271; Biolegend) for isolation of swMBCs.
Instrument	Flow cytometry analyses were performed using a LSR-II flow cytometer (BD Biosciences) or FACSCelesta flow cytometer (BD Biosciences). FACS sorting were performed using a FACSAria-III cell sorter (BD Biosciences).
Software	Flow cytometry data were analyzed using FlowJo software (TreeStar).
Cell population abundance	Human B cells were purified using the EasySep™ Human Total B Cell Isolation Kit (STEMCELL Technologies) before FACS sorting. The purity of these B cells were >99%, as analyzed for CD19+ cells by flow cytometry. Among the total B cells, about 75-79% were IgD+CD27- naive B cells, 7.7% were IgD+CD27+ memory B cells, 3.3% were IgG+CD27+ memory B cells and 2.6% were IgA +CD27+ memory B cells.
Gating strategy	FACS analyses were performed on single cell suspensions. In all flow cytometry experiments, cells were appropriately gated by forward and side-scattering to exclude dead cells and debris.

- [x] Tick this box to confirm that a figure exemplifying the gating strategy is provided in the Supplementary Information.
